# Monkeypox virus protein H3L induces injuries in human and mouse

**DOI:** 10.1038/s41419-024-06990-2

**Published:** 2024-08-21

**Authors:** Shaoxian Chen, Guiping Huang, Juli Liu

**Affiliations:** 1 Medical Research Institute, Guangdong Provincial People’s Hospital (Guangdong Academy of Medical Sciences), Southern Medical University, Guangzhou, China; 2 Guangdong Provincial Key Laboratory of South China Structural Heart Disease, Guangdong Provincial People’s Hospital, Guangdong Academy of Medical Sciences, Guangzhou, China; 3 Guangdong Cardiovascular Institute, Guangdong Provincial People’s Hospital (Guangdong Academy of Medical Sciences), Guangzhou, Guangdong China

**Keywords:** Viral infection, Cardiovascular diseases, Experimental models of disease, Infection, Stem-cell research

## Abstract

Monkeypox virus (MPV) is known to inflict injuries and, in some cases, lead to fatalities in humans. However, the underlying mechanisms responsible for its pathogenicity remain poorly understood. We investigated functions of MPV core proteins, H3L, A35R, A29L, and I1L, and discovered that H3L induced transcriptional perturbations and injuries. We substantiated that H3L upregulated IL1A expression. IL1A, in consequence, caused cellular injuries, and this detrimental effect was mitigated when countered with IL1A blockage. We also observed that H3L significantly perturbed the transcriptions of genes in cardiac system. Mechanistically, H3L occupied the promoters of genes governing cellular injury, leading to alterations in the binding patterns of H3K27me3 and H3K4me3 histone marks, ultimately resulting in expression perturbations. In vivo and in vitro models confirmed that H3L induced transcriptional disturbances and cardiac dysfunction, which were ameliorated when IL1A was blocked or repressed. Our study provides valuable insights into comprehensive understanding of MPV pathogenicity, highlights the significant roles of H3L in inducing injuries, and potentially paves the way for the development of therapeutic strategies targeting IL1A.

## Introduction

The disease mpox (monkeypox) is a viral illness caused by the monkeypox virus (MPV), which is an enveloped double-stranded DNA virus of the *Orthopoxvirus* genus in the *Poxviridae* family [1]. MPV was first discovered in monkeys, but evidence show that MPV can be transmitted to humans through direct contact with infected animals [1]. It can also spread from person to person through respiratory droplets or close contact with infected individuals [1, 2]. MPV infection typically includes fever, muscle aches, and a rash [3]. However, severe complications can occur, such as pneumonia, encephalitis (inflammation of the brain), cardiac complications [2–7], and even death [1], Since early May 2022, cases of MPV infection had been reported from countries where the disease is not endemic, and continued to be reported in several endemic countries [8]. The global outbreak already caused more than 89,000 cases and 150 deaths reported to WHO from 111 countries. Although it was no longer a Public Health Emergency of International Concern (PHEIC), according to a statement by the World Health Organization on May 2023, MPV continues to circulate and will be a thread for human. Consequently, the aftermath of MPV infection in humans necessitates further comprehensive investigation to better comprehend its implications.

There are currently no specific antiviral vaccines for MPV infection. Although smallpox vaccines (such as the ACAM2000 licensed by the U.S. Food and Drug Administration) may have some efficacy in preventing MPV [9], the degree and durability of such protection was unknown because of the limited real-world MPV vaccine performance data [9]. Regarding other treatments rather than vaccines, there are no specific drugs targeting MPV as well. Although several potential drugs were reported to treat MPV infection [10], they are still in clinical trials and not approved for human. The fact that there is currently no specific drugs for MPV is attributed to its unclear pathogenesis in human [11]. Thus, studying MPV induced-injuries and delineating underlying molecular mechanisms in human is critical and urging for vaccine and drug development.

The MPV genome encodes about 200 proteins, many are membrane proteins. MPV contains over 30 structural and membrane viral proteins as well as some virus-encoded DNA-dependent RNA polymerase and associated transcriptional enzymes, which have potential DNA binding activities [12, 13]. MPV can use membrane proteins to interact with host cells, causing virus infection or cell injuries [14]. MPV may enter host cells by either fusion with the plasma membrane or endocytosis, and at least 16 proteins in the virus membrane are involved in the entry process [15]. After entry, the virus initiates early gene transcription events and viral DNA synthesis by using its proteins with DNA binding activities, such DNA and RNA polymerases [16, 17]. The virus membrane protein interaction with human cells, the viral early gene transcription and DNA synthesis may cause injuries [18]. Thus, MPV proteins on membrane or having DNA binding activities could be potential drug and vaccine targets for preventing infection or injuries [19]. However, the functions of most MPV proteins are unclear and remain to be urgently elucidated before the specific drugs or vaccines will be designed.

To comprehensively understand which MPV protein is pathogenic and directly causes injuries in human, and what is the underlying mechanism in the injuries, we initially established an in vitro system of human embryonic stem cells (hESCs) model, overexpressed 4 of the MPV core proteins in hESCs. In the in vitro system, we conducted a screening and identified that two MPV core proteins (H3L and A29L) were pathogenic and directly induced transcriptional perturbations, resulting in DNA damage and cell death. Furthermore, we found that H3L induced cellular injuries via activating IL1A, which could be mitigated by IL1A blockage. Interestingly, we observed that H3L caused transcriptional perturbations in cardiac system. Moreover, H3L induced cardiac injuries in vitro and in vivo, which could be attenuated by IL1A blockage or repression. Mechanistically, H3L could bind to promoters of cardiac genes, leading to binding alterations of H3K27me3 and H3K4me3 on the promoters, which finally cause expression perturbations.

In this study, we established a platform to delineate MPV pathogenesis in human and mouse. Our findings demonstrated that Monkeypox virus (MPV) protein (H3L) directly caused perturbations of gene expression in both human and mouse, resulting in cellular injuries. These injuries were attributed to their ability to activate or repress normal transcriptions by binding to promoters and re-modeling the chromatin status. Our findings also highlighted that H3L and IL1A may be potential therapeutic targets for MPV-associated injuries.

## Results

### Two MPV proteins, H3L and A29L, induce transcriptional perturbations and DNA damage in human embryonic stem cells (hESCs)

**Fig. 1:**
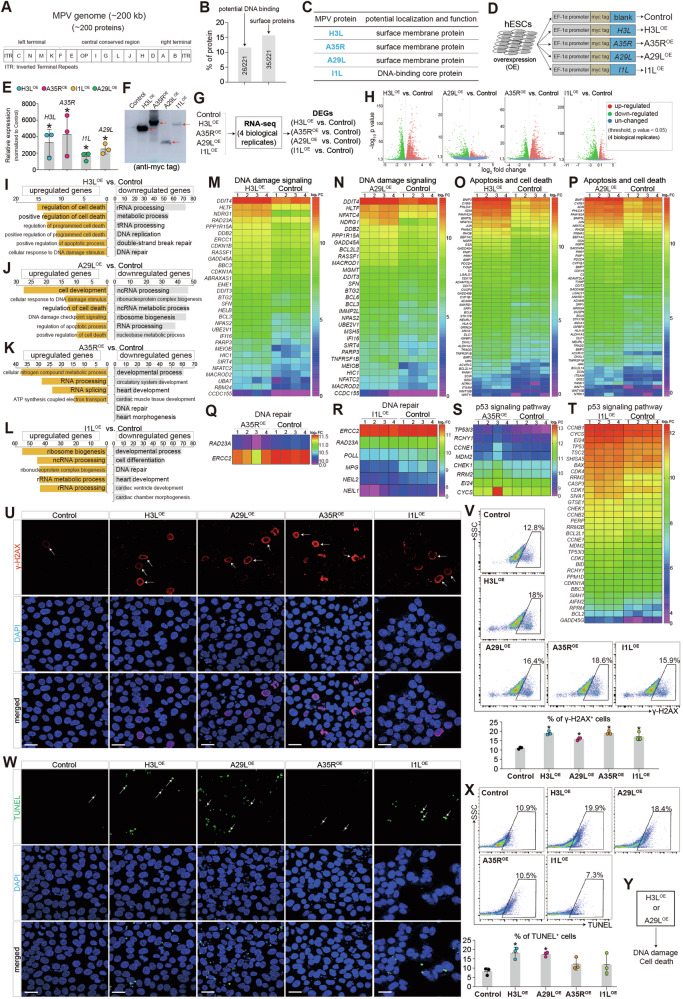
Two MPV proteins, H3L and A29L, induce transcriptional perturbations and DNA damage in human embryonic stem cells (hESCs). **A** The structure of Monkeypox virus (MPV) genome. **B** Classification of MPV proteins. **C** 4 of core proteins, H3L, A35R, A29L, and I1L, in MPV. **D** Overexpression of H3L, A35R, A29L and I1L in human embryonic stem cells (hESCs) using lentivirus. H3L, A35R, A29L, and I1L were fused with myc tag on N terminus. OE, overexpression. **E** RT-qPCR showing the relative expression levels of H3L, A35R, A29L, and I1L (normalized to Control). *p < 0.01 (vs. Control). **F** Western-blotting showing the protein expression levels of Control, H3L, A35R, A29L and I1L in hESCs. **G** RNA-seq analysis of Control, H3L^OE^, A35R^OE^, A29L^OE^ and I1L^OE^ hESCs. Four biological replicates were applied. DEGs, differentially expressed genes. **H** Volcano plots showing the differentially expressed genes (DEGs). P < 0.05 and |log_2_(fold change)| > 0 were set as the threshold for DEGs. **I**–**L** Gene Ontology (GO) analyses of differentially expressed genes. **M**–**T** Heatmap showing differentially expressed genes in signature events. **U** Immunostaining of γ-H2AX in Control, H3L^OE^, A35R^OE^, A29L^OE^ and I1L^OE^ hESCs. Red color showed γ-H2AX positive (γ-H2AX^+^) signal. Blue color showed DAPI (nucleus). Scale bar, 50 µm. **V** Flow cytometry quantification of γ-H2AX^+^ cells. *p < 0.05 (vs. Control). **W** Immunostaining of TUNEL in Control, H3L^OE^, A35R^OE^, A29L^OE^ and I1L^OE^ hESCs. Green color showed TUNEL positive (TUNEL^+^) signal. Blue color showed DAPI (nucleus). Scale bar, 50 µm. **X** Flow cytometry quantification of TUNEL^+^ cells. *p < 0.05 (vs. Control). **Y** The function of H3L and A29L in hESCs.

The MPV genome is linear and approximately 197 kb in length and encodes about 200 proteins. It contains a central region flanked by two ITRs (Inverted Terminal Repeats), resulting in a unique hairpin structure at each end (Fig. 1A). MPV infection can lead to damages in human tissues, but the specific gene responsible for these injuries remains unknown. MPV proteins include surface membrane proteins, RNA polymerase and some proteins having potential DNA binding ability (Fig. 1B, C, Fig. S1A), which could serve as potential vaccine or drug targets [20–22]. MPV has more than 10 core proteins [23, 24]. The surface membrane proteins, A29L, A35R and H3L, are three of the core proteins (The genome of MPV ON563414 in NCBI). H3L is similar to Vaccinia virus strain Copenhagen H3L heparin binding surface protein (Cop-H3L) surface membrane protein. A35R is the envelope glycoprotein, needed for formation of actin-containing microvilli and cell-to-cell spread of virion. A29L is surface membrane, binding to cell surface heparan similar to Vaccinia virus strain. I1L is the DNA-binding core protein (Cop-I1L), which is similar to Vaccinia virus strain Copenhagen I1L virosomal protein and essential for virus multiplication. These proteins are important because surface membrane proteins or viral replication-associated proteins could be potential vaccine or drug targets [25, 26]. However, the pathogenesis of A29L, A35R, H3L and I1L are unclear.

To investigate the functions of these proteins, we overexpressed them individually in human embryonic stem cells (hESCs) tagged with myc (H3L^OE^, A35R^OE^, A29L^OE^ and I1L^OE^ hESCs) (Fig. 1D, Fig. S1B, C). At both RNA (Fig. 1E, Fig. S1D) and protein (Fig. 1F, Fig. S1E) levels, H3L, A35R, A29L and I1L were expressed in H3L^OE^, A35R^OE^, A29L^OE^ and I1L^OE^ hESCs, respectively. We applied bulk RNA-seq on Control, H3L^OE^, A35R^OE^, A29L^OE^ and I1L^OE^ hESCs (Fig. 1G) and found that H3L, A35R, A29L and I1L induced transcriptional perturbations (Fig. 1H, Fig. S1F). We found that H3L- and A29L-upregulated genes were involved into cell death and DNA damage (Fig. 1I, J, Fig. S1G, H), whereas A35R- and I1L-upregulated genes might regulate RNA processing (Fig. 1K, L, Fig. S1I, J). Specifically, both H3L and A29L significantly increased the expression of genes in DNA damage (Fig. 1M, N) and genes in apoptosis/cell death (Fig. 1O, P). We observed that A35R and I1L regulated gene expression involved in DNA repair and P53 signaling pathway (Fig. 1Q–T). To verify this, we evaluated the DNA damage by detecting γ-H2AX (an early cellular response to DNA double-strand breaks) and TUNEL (a marker for DNA damage and cell death) in hESCs overexpressing H3L, A35R, A29L, and I1L. Data showed that H3L, A35R, A29L and I1L increased percentage of γ-H2AX^+^ cells (Fig. 1U, V). However, only H3L and A29L, not A35R and I1L, increased percentage of TUNEL^+^ cells (Fig. 1W, X). The evidence demonstrated that the two MPV proteins, H3L and A29L, induced transcriptional perturbations, resulting in injuries in hESCs (Fig. 1Y).

### H3L induces DNA damage via upregulating IL1A in human

**Fig. 2:**
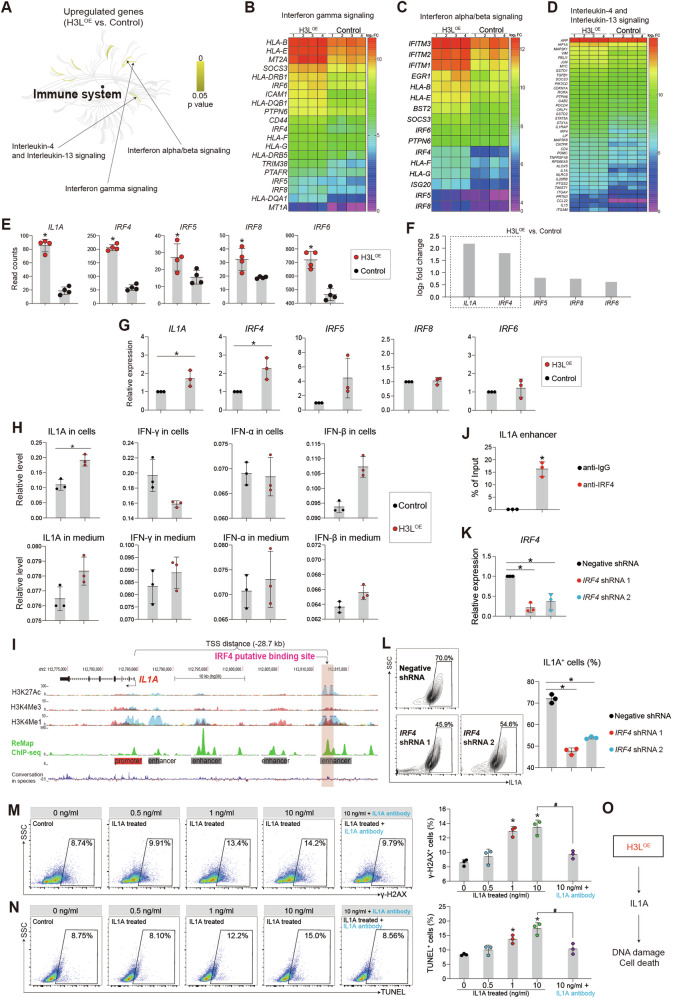
H3L induces DNA damage via upregulating IL1A. **A** Signaling pathway enrichment analyses of differentially expressed genes. Enrichment analyses were run on Reactome. Heatmap showing differentially expressed genes involved in interferon signaling pathway (**B**–**D**) and interleukin signaling pathway (**D**). **E** RNA-seq read counts of signature genes involved in interferon and interleukin signaling pathways. Four biological replicates were applied for RNA-seq. *p < 0.05 (vs. Control). **F** Relative expression levels of signature genes involved in interferon and interleukin signaling pathways in RNA-seq. **G** RT-qPCR showing relative expression levels of signature genes involved in interferon and interleukin signaling pathways. *p < 0.05 (vs. Control). **H** The enzyme-linked immunosorbent assay (ELISA) showing relative expression levels of IL1A, IFN-γ, IFN-α, and IFN-β. *p < 0.05 (vs. Control). Relative level in the Y-axis meant the read count on the absorption at 450 nm by the equipment. **I** Representative ChIP-seq peaks on human *IL1A*. There was a potential binding site on upstream distal enhancer of *IL1A* showed by ChIP-seq. TSS, transcriptional start site. Data was from the published datasets in UCSC genome browser. **J** ChIP-qPCR showing IRF4 enrichment on the binding site on upstream distal enhancer of *IL1A* in WT hESCs. *p < 0.05 (vs. IgG). **K** RT-qPCR showing the expression of *IRF4* in negative control and shRNAs knockdown hESCs. *p < 0.05 (vs. Negative shRNA control). **L** Flow cytometry quantification of IL1A^+^ cells in negative control and *IRF4*-shRNA knockdown hESCs. *p < 0.05 (vs. Negative shRNA control). **M** Flow cytometry quantification of γ-H2AX^+^ hESCs treated with different concentration of IL1A. IL1A antibody was used to block IL1A activity. *p < 0.05 (vs. 0 ng/ml), ^#^p < 0.05. **N** Flow cytometry quantification of TUNEL^+^ hESCs treated with different concentration of IL1A. IL1A antibody was used to block IL1A activity. *p < 0.05 (vs. 0 ng/ml), ^#^p < 0.05. **O** Working model of H3L-driven IL1A in inducing DNA damage and cell death.

H3L exhibited a pronounced enhancement of DNA damage (Fig. 1U–X). Additionally, we found that H3L overexpression caused genome instability (Fig. S1K), increased the expression levels of apoptotic markers (Fig. S1L–N). Consequently, our research is directed towards investigating the specific roles played by H3L. The enrichment analyses of RNA-seq revealed that H3L activated Interferon and Interleukin-4/Interleukin-13 signaling pathways (Fig. 2A–D, Fig. S2A). Particularly, *IL1A* was among the top-ranked upregulated genes, and it was highly associated with upregulations of several interferons regulatory factors (IRFs), such as *IRF4*, *IRF8* and *IRF6* (Fig. 2E, F). RT-qPCR confirmed that *IL1A* and *IRF4* were significantly up-regulated in H3L^OE^ hESCs (Fig. 2G). At protein level, IL1A was significantly increased after H3L overexpression (Fig. 2H). IRFs were transcriptional factors and reported to induce apoptosis and cell death [27, 28]. This evidence suggests that H3L might induce cell injuries through the IRF4-IL1A signaling pathway.

We observed that *IRF4* and *IL1A* had similar expression pattern (Fig. 2F) and they were concurrently up-regulated in H3L^OE^ hESCs (Fig. 2E–G). This led us to hypothesize that IRF4, functioning as a transcription factor [27, 28], might potentially promote the transcription of *IL1A*. Supporting this notion, we identified a potential binding site for IRF4 on the distal enhancer of *IL1A*, where the presence of H3K4me1, H3K4me3, and H3K27Ac marks suggested active regulatory elements (Fig. 2I, Fig. S2B). To verify this, ChIP-qPCR confirmed that IRF4 occupied the potential binding site (Fig. 2J). Furthermore, knockdown of IRF4 by shRNAs (Fig. 2K) significantly down-regulated IL1A protein expression (Fig. 2L). Thus, the evidence demonstrated that IRF4 promoted *IL1A* expression via directly binding to *IL1A* distal enhancer.

IL1A was reported to induce cell injuries, including inflammation and cell death, in animal [29, 30]. However, whether IL1A upregulation driven by H3L could cause cell injuries in human is unclear. We treated hESCs with IL1A and found that IL1A could increase the expression of γ-H2AX (Fig. 2M) and TUNEL (Fig. 2N). However, when an antibody to block IL1A was applied, the IL1A-induced DNA damage was significantly reduced (Fig. 2M, N). This confirmed that IL1A played a role in causing DNA damage. Taken together, we demonstrated that H3L induces DNA damage via upregulating IL1A in human (Fig. 2O).

### H3L induces cellular injuries via IL1A upregulation in human cardiac lineages

**Fig. 3:**
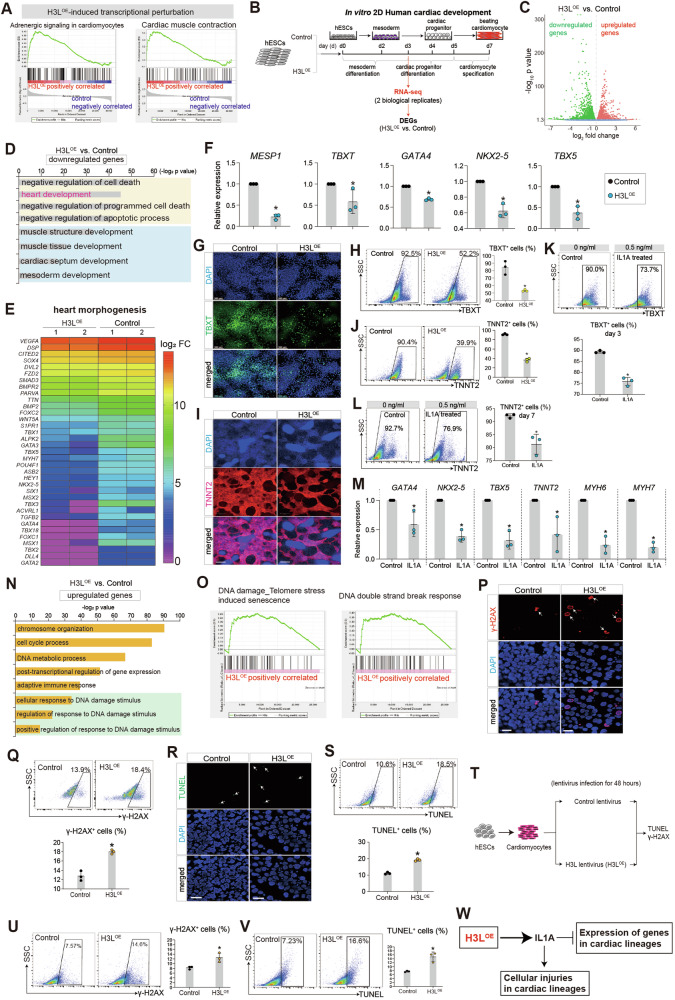
H3L induces cellular injuries via IL1A upregulation in human cardiac lineages. **A** Gene Set Enrichment Analysis (GSEA) of RNA-seq data showing the transcriptional perturbations induced by H3L on cardiac genes in hESCs. RNA-seq data was from Fig. 1G. **B** RNA-seq analysis of cardiac lineage cells on day 3 (d3) of differentiation. Two biological replicates were applied. DEGs, differentially expressed genes. **C** Volcano plots showing differentially expressed genes (DEGs) induced by H3L. P < 0.05 and |log_2_(fold change) | > 0 were set as the threshold for DEGs. **D** Gene Ontology (GO) analysis of downregulated genes induced by H3L. **E** Heatmap showing H3L-downregulated genes involved in heart morphogenesis and cardiac development. FC, fold change. **F** RT-qPCR showing gene expression changes in cardiac lineage cells of day 3. *p < 0.05 (vs. Control). **G** Immunostaining of TBXT^+^ cells in cardiac lineage cells of day 3. Green showed TBXT. Blue showed DAPI. Scale bar, 100 µm. **H** Flow cytometry quantification of TBXT ^+^ cells in cardiac lineage cells of day 3 from (**G**). *p < 0.05 (vs. Control). **I** Immunostaining of TNNT2^+^ cells in cardiac lineage cells of day 7. Red showed TNNT2. Blue showed DAPI. Scale bar, 200 µm. **J** Flow cytometry quantification of TNNT2 ^+^ cells in cardiac lineage cells of day 7 from (**I**). *p < 0.05 (vs. Control). **K** Flow cytometry quantification of TBXT ^+^ cells in cardiac lineage cells of day 3. IL1A was added from day 0 to day 3 during cardiac differentiation. IL1A final concentration was 0.5 ng/ml. *p < 0.05 (vs. 0 ng/ml Control). **L** Flow cytometry quantification of TNNT2 ^+^ cells in cardiac lineage cells of day 7. IL1A was added from day 0 to day 7 during cardiac differentiation. IL1A final concentration was 0.5 ng/ml. *p < 0.05 (vs. 0 ng/ml Control). **M** RT-qPCR showing relative expression of cardiogenic genes in cardiac lineage cells of day 7. IL1A was added from day 0 to day 7 during cardiac differentiation. IL1A final concentration was 0.5 ng/ml. Control, no IL1A. *p < 0.05 (vs. Control). **N** Gene Ontology (GO) analysis of upregulated genes induced by H3L. **O** GSEA analysis showing senescence and DNA damage signaling pathways. **P** Immunostaining of γ-H2AX^+^ cells in cardiac lineage cells of day 3. Red showed γ-H2AX. Blue showed DAPI. Scale bar, 200 µm. **Q** Flow cytometry quantification of γ-H2AX ^+^ cells in cardiac lineage cells of day 3 from (**P**). *p < 0.05 (vs. Control) (**R**) Immunostaining of TUNEL^+^ cells in cardiac lineage cells of day 3. Green showed TUNEL. Blue showed DAPI. Scale bar, 200 µm. **S** Flow cytometry quantification of TUNEL^+^ cells in cardiac lineage cells of day 3. *p < 0.05 (vs. Control). **T** Human cardiomyocytes derived from hESCs were infected with lentiviruses to overexpress H3L, followed with quantification of γ-H2AX^+^ and TUNEL^+^ cells on 48 h later. Blank virus infection was used as Control. Flow cytometry quantification of γ-H2AX^+^ (**U**) and TUNEL^+^ (**V**) cardiomyocytes. *p < 0.05 (vs. Control). **W** H3L induces cellular injuries in human cardiac lineages via IL1A.

Enrichment analyses revealed that the presence of H3L led to significant transcriptional disturbances in cardiac genes in hESCs (Fig. 3A), which indicated that H3L might play a role in cardiac system. To further investigate it, we differentiated Control and H3L^OE^ hESCs into mesodermal and cardiac lineages (Fig. 3B). Subsequent RNA-seq analysis of the human cardiac lineages (day 3 post differentiation) demonstrated that H3L had a global impact on transcription, inducing significant perturbations (Fig. 3C, Fig. S3A, B). Enrichment analyses revealed that H3L significantly down-regulated genes, which were involved in heart morphogenesis and cardiac muscle development (Fig. 3D, E, Fig. S3C, D). RT-qPCR (Fig. S3E) confirmed that H3L resulted in decreased expression levels of critical mesodermal inducers (*MESP1, TBXT*) and cardiogenic genes (*GATA4, NKX2-5, TBX5*) (Fig. 3F). At protein levels, H3L decreased percentage of TBXT^+^ cells (Fig. 3G, H). Moreover, H3L decreased percentage of TNNT2^+^ cells (Fig. 3I, J). The evidence demonstrated that H3L disrupted transcriptions in cardiac system, resulting in inhibition of human cardiac development in hESCs, including mesoderm differentiation and cardiomyocyte specification.

Our data showed that IL1A, driven by H3L, directly promoted DNA damage in hESCs (Fig. 2M, N). However, whether the repression of cardiac gene in H3L^OE^ cells (Fig. 3A–K) was attributed to the injuries indued by IL1A (Fig. 2M, N) was unclear. To confirmed it, we treated hESCs with IL1A during cardiac development (Fig. 3B). We found that IL1A treatment significantly decreased percentage of TBXT^+^ (Fig. 3K) and TNNT2^+^ (Fig. 3L) cells. Additionally, RT-qPCR data showed that IL1A significantly repressed expression levels of cardiogenic transcription factors (*GATA4*, *NKX2-5*, *TBX5*) and cardiomyocyte-specific markers (*TNNT2, MYH6, MYH7*) (Fig. 3M). This confirmed the abnormal expression patterns of cardiac genes induced by H3L-driven IL1A upregulation.

H3L overexpression also up-regulated genes involved in senescence, and DNA damage (Fig. 3N, O, Fig. S3F, G). Additionally, our data showed that H3L^OE^ significantly increased percentages of γ-H2AX^+^ cells (Fig. 3P, Q) and TUNEL^+^ cells (Fig. 3R, S) (day 3 post differentiation). We also infected hESC-derived cardiomyocytes with Control and H3L lentivirus and evaluated the expression of γ-H2AX and TUNEL (Fig. 3T). We found that H3L could directly promote DNA damage in cardiomyocytes (Fig. 3U, V). Taken together, our findings demonstrated that H3L induces cellular injuries and represses cardiac genes via IL1A upregulation in human cardiac lineages (Fig. 3W).

### H3L occupies promoters to re-model H3K27me3 and H3K4me3, resulting in expression perturbations

**Fig. 4:**
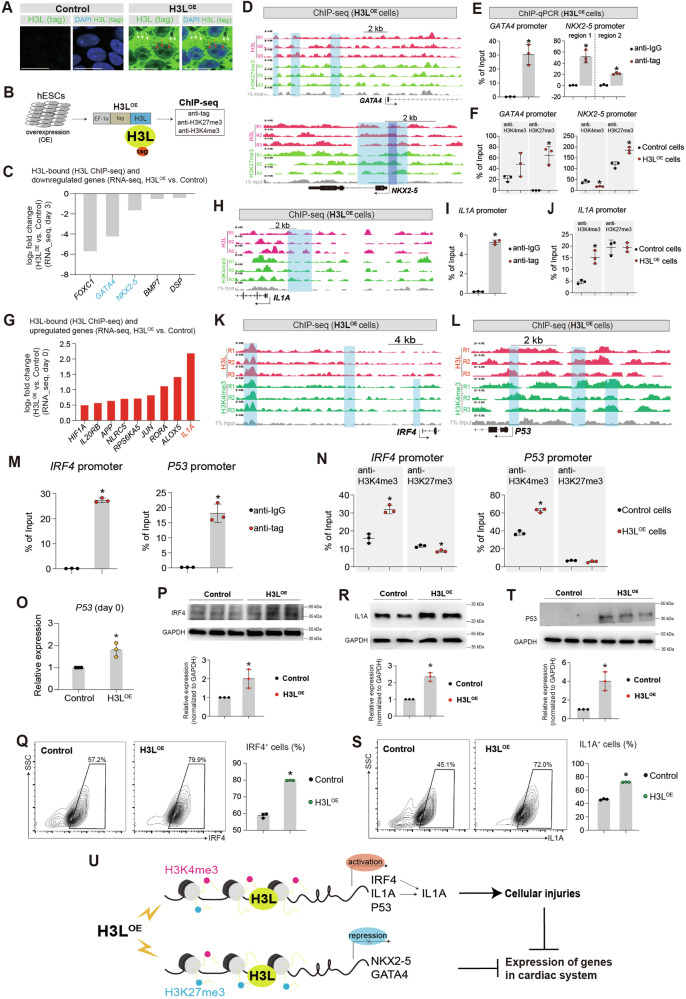
H3L occupies promoters to re-model H3K27me3 and H3K4me3, resulting in expression perturbations. **A** Immunostaining showing the expression and localization of H3L in hESCs. Green showed H3L protein expression (tag expression). Blue showed DAPI (nucleus). White arrows showed H3L protein localized in cytoplasm. Red arrows showed H3L protein localized in nucleus. Scale bar, 20 µm. **B** Chromatin Immunoprecipitation (ChIP) assays followed with sequencing (ChIP-seq) on H3L^OE^ hESCs. H3L were fused with myc-tag on N terminus. Anti-myc tag can enrich H3L-bound chromatin. H3K27me3 is an indicator of repressive transcription. H3K4me3 is an indicator of active transcription. Three biological replicates were applied. **C** RNA-seq (from Fig. 3B) showing the downregulated genes (H3L^OE^ vs. Control), which were bound by H3L in ChIP-seq. **D** Representative ChIP-seq peaks showing binding of H3L on cardiogenic transcription factor (*GATA4, NKX2-5*) in H3L^OE^ hESCs. Blue boxes highlighted binding regions of H3L or H3K27me3. **E** ChIP-qPCR validation of H3L binding on promoters of *GATA4* and *NKX2-5* in H3L^OE^ hESCs. *p < 0.05 (vs. anti-IgG). **F** ChIP-qPCR showing the binding changes of H3K4me3 and H3K27me3 on promoters of *GATA4* and *NKX2-5* between Control and H3L^OE^ hESCs. *p < 0.05 (H3L^OE^ cells vs. Control cells). **G** RNA-seq (from Fig. 1) showing the upregulated genes (H3L^OE^ vs. Control), which were bound by H3L in ChIP-seq. **H** Representative ChIP-seq peaks showing binding of H3L on *IL1A* promoter in H3L^OE^ hESCs. Blue boxes highlighted binding regions of H3L or H3K4me3. **I** ChIP-qPCR validation of H3L binding on *IL1A* promoter in H3L^OE^ hESCs. *p < 0.01 (vs. anti-IgG). **J** ChIP-qPCR showing the binding changes of H3K4me3 and H3K27me3 on *IL1A* promoter between Control and H3L^OE^ hESCs. *p < 0.05 (H3L^OE^ cells vs. Control cells). **K** Representative ChIP-seq peaks showing binding of H3L on upstream regions of *IRF4* transcription start site in H3L^OE^ hESCs. Blue boxes showed binding regions of H3L or H3K4me3. **L** Representative ChIP-seq peaks showing binding of H3L on *P53* promoter in H3L^OE^ hESCs. Blue boxes showed binding regions of H3L or H3K4me3. **M** ChIP-qPCR showing the binding of H3L on *IRF4* promoter (left) and *P53* promoter (right) in H3L^OE^ hESCs. *p < 0.01 (anti-IgG vs. anti-tag). **N** ChIP-qPCR showing the binding changes of H3K4me3 and H3K27me3 on promoters of *IRF4* (left) and *P53* (right) between Control and H3L^OE^ hESCs. *p < 0.05 (H3L^OE^ cells vs. Control cells). **O** RT-qPCR showing relative expression level of *P53* in hESCs (day 0). p* < 0.05 (vs. Control). **P** Western-blot showing IRF4 protein expression in Control and H3L^OE^ hESCs. GAPDH, an internal control. *p < 0.05 (vs. Control). **Q** Flow cytometry quantification of IRF4^+^ cells in Control and H3L^OE^ hESCs. *p < 0.01 (vs. Control). **R** Western-blot showing IL1A protein expression in Control and H3L^OE^ hESCs. GAPDH, an internal control. *p < 0.05 (vs. Control). **S** Flow cytometry quantification of IL1A^+^ cells in Control and H3L^OE^ hESCs. *p < 0.01 (vs. Control). **T** Western-blot showing P53 protein expression in Control and H3L^OE^ hESCs. GAPDH, an internal control. *p < 0.05 (vs. Control). **U** The working model of molecular mechanisms in H3L-induced injuries.

We discovered that H3L significantly perturbs the expression levels of epigenetic regulators (Fig. S4A–C). Furthermore, our observations revealed that although H3L primarily localizes in the cytoplasm, it also partially localizes in the nucleus (Fig. 4A, Fig. S4D, E). Additionally, H3L increased the protein expression of histone 3 (Fig. S4F). This suggests that the nuclear presence of H3L might directly impact gene transcription through both epigenetic regulation and the remodeling of histone 3. To investigate this further, we applied ChIP-seq on H3L^OE^ hESCs using myc-tag, H3K27me3 and H3K4me3 antibodies (Fig. 4B, Fig. S5A–D). We found that H3L could bind genes associated with heart development (Fig. S5E, F). We overlapped RNA-seq and ChIP-seq and found that some genes controlling cardiac development were bound and downregulated by H3L (Fig. 4C, Fig. S6A–D). Specifically, The ChIP-seq results uncovered that H3L bound to the promoters of cardiogenic inducers/TFs (*GATA4, NKX2-5*) (Fig. 4D, E), which were significantly repressed by H3L overexpression (Fig. 4C, Fig. S6A). These regions exhibited enrichment of H3K27me3 (Fig. 4D), a marker of transcriptional repression [31]. To corroborate these findings, we performed ChIP-qPCR and confirmed the occupancy of their promoters by H3L in H3L^OE^ cells (Fig. 4E), which resulted in increased H3K27me3 binding and reduced H3K4me3 binding on these promoters in H3L^OE^ cells, compared to Control cells (Fig. 4F). This provides evidence that the expression perturbations of cardiac genes (Figs. 3, 4C) were attributed to H3L binding to these promoters and altering the methylation status of lysine 27 or lysine 4 on histone 3. This, in turn, results in the repression of cardiac lineage specifications (Fig. 3).

We overlapped RNA-seq and ChIP-seq and found that some genes associated with cell death were bound and upregulated by H3L (Fig. 4G, Fig. S6E). Specifically, ChIP-seq unveiled that H3L occupied promoters of *IL1A* (Fig. 4H), which was validated by ChIP-qPCR (Fig. 4I). We found that H3L occupancy on *IL1A* promoter led to increased binding of H3K4me3 on the promoter (Fig. 4J), thereby contributing to upregulation of *IL1A* (Fig. 4G). Even though *IRF4* and *P53* were not among the genes with overlapping results (Fig. 4G), we observed that H3L could occupy upstream regions of *IRF4* transcription start site (TSS) and *P53* promoter (Fig. 4K–M). The binding of H3L to these upstream regions resulted in increased H3K4me3 binding and reduced H3K27me3 binding within these regions (Fig. 4N). These findings elucidate the significantly elevated RNA expression levels of IRF4 and P53 due to H3L (Fig. 2, Fig. 4O, Fig. S6F–I). This upregulation was also reflected in the protein expression levels of IRF4 and P53 (Fig. 4P–T). These observations collectively suggest that H3L-induced upregulation of IL1A contributes to cellular injuries. Moreover, the upregulation of P53 by H3L may also play a role in inducing cellular injuries, as P53 is well-established in its function of promoting cell death [32–36].

In summary, our findings collectively demonstrate that H3L has a dual impact: (1) it inhibits the expression of genes in the cardiac system by binding to the promoters of cardiogenic inducers (such as *GATA4, NKX2-5*) and (2) it induces cellular injuries through the activation of IL1A and P53 pathways (Fig. 4U). Mechanistically, H3L’s occupation of promoters and its modification of H3K27me3 and H3K4me3 on these promoters result in expression perturbations (Fig. 4U).

### H3L induces transcriptional perturbations in mouse heart tissues

**Fig. 5:**
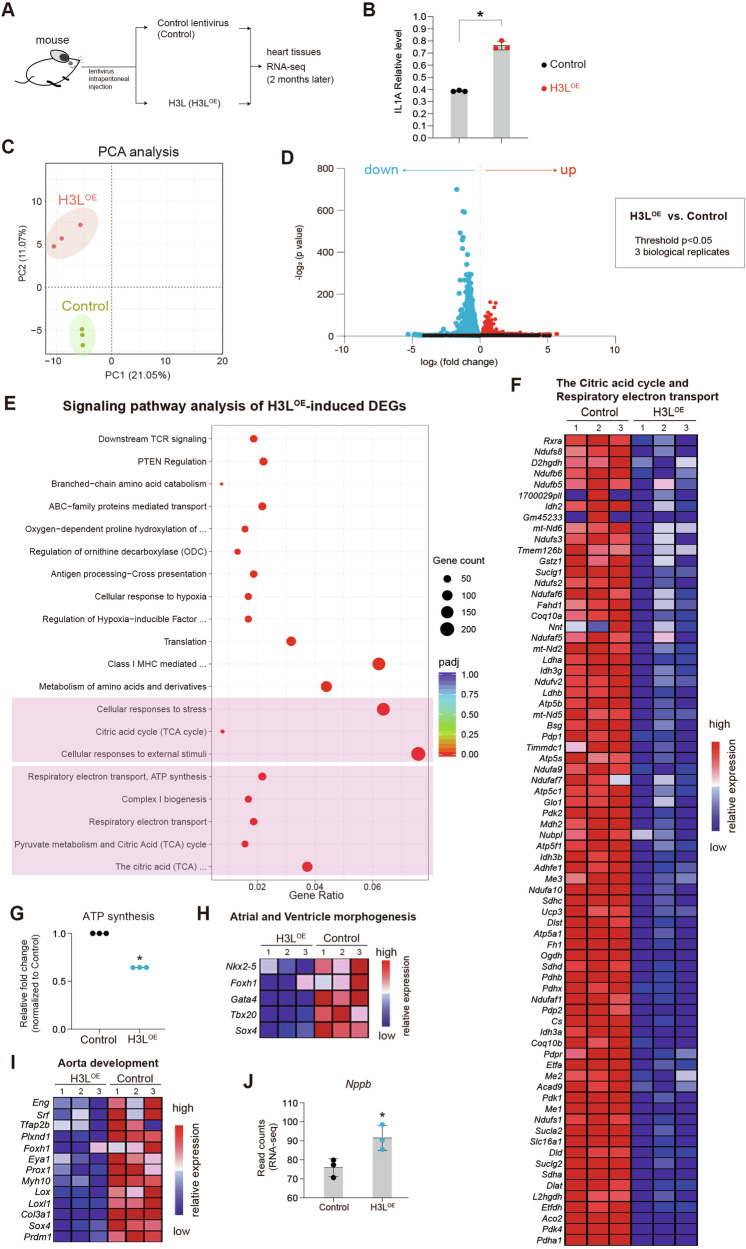
H3L induce transcriptional perturbations in mouse heart. **A** Scheme of in vivo mouse model to study the effects of H3L in heart. Lentiviruses with control and H3L^OE^ were intraperitoneally injected into one month old mouse. Two months later, heart tissues were collected for bulk RNA-seq. **B** ELISA assay showing protein expression level of IL1A in blood plasma from mouse heart. *p < 0.05 (vs. Control). Relative level in the Y-axis meant the read count on the absorption at 450 nm by the equipment. **C** Principal component analysis (PCA) of RNA-seq on mouse heart tissues. Three biological replicates were applied for RNA-seq. **D** Volcano plots showing differentially expressed genes (DEGs) in heart tissues induced by H3L. P < 0.05 and | log_2_(fold change) | > 0 were set as the threshold for DEGs. **E** Signaling pathway analysis of differentially expressed genes induced by H3L. The top 20 of highest ranked GO terms were presented. Pathway analysis was run on Reactome. Padj, adjusted *p* value. **F** Heatmap showing differentially expressed genes (DEGs) induced by H3L, which were involved in the Citric acid cycle and Respiratory electron transport. **G** Evaluation of ATP amount in mouse neonatal cardiomyocytes overexpressed with control lentivirus (Control) or H3L lentivirus (H3L^OE^). *p < 0.05 (vs. Control). Heatmap showing differentially expressed genes induced by H3L, which were involved in the atrial/ventricle morphogenesis (**H**) and aorta development (**I**). **J** RNA-seq read counts showing the expression levels of cardiac hypertrophy marker *Nppb* in Control and H3L^OE^ mouse heart tissues.

Our findings demonstrated that H3L induced transcriptional perturbations and injuries in human cardiac lineages (Fig. 3), we therefore sought to assess whether these effects could also be observed in the mouse heart in vivo (Fig. 5A, Fig. S7A). Interestingly, similar to the observed phenotype of IL1A upregulation in hESCs (Fig. 2), H3L also significantly up-regulated the protein expression level of Il1a in the blood plasma from the mouse heart (Fig. 5B). RNA-seq analysis revealed that H3L caused changes in the expression patterns of genes in mouse heart (Fig. 5C, D). Enrichment analyses of the differentially expressed genes (DEGs) induced by H3L^OE^ showed involvement in the Citric acid cycle and respiratory electron transport/ATP synthesis (Fig. 5E, Fig. S7B), further analysis showed that H3L^OE^ decreased expression levels of genes in respiratory electron transport/ATP synthesis (Fig. 5F). Furthermore, we found ATP synthesis was significantly inhibited by H3L overexpression (Fig. 5G). This demonstrated that H3L resulted in the impairment of oxidative phosphorylation and ATP biosynthesis in mouse heart tissues. Although the cardiovascular events were not among the top-ranked events, H3L overexpression down-regulated expression levels of cardiovascular genes, including genes controlling atrial/ventricle morphogenesis (Fig. 5H) and aorta development (Fig. 5I). We observed that genes governing collagen formation (Fig. S7C, D), as well as the cardiac hypertrophic marker *Nppb* (Fig. 5J), were upregulated by H3L in mouse heart tissues. These findings indicated that H3L induced transcriptional perturbations in mouse heart tissues, potentially resulting in cardiac remodeling such as hypertrophy and fibrosis.

### H3L induces cardiac injuries in mouse, which are mitigated by blocking IL1A

**Fig. 6:**
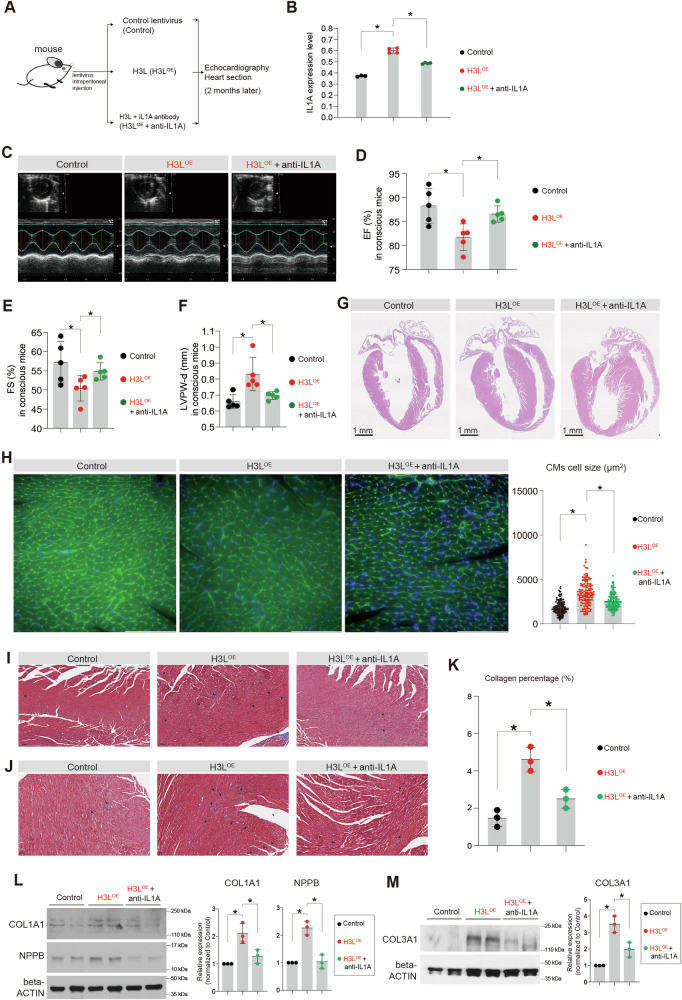
H3L induces cardiac remodeling in mouse, which are attenuated by IL1A blockage. **A** Scheme of in vivo mouse model to study the effects of H3L in mouse heart tissues. Lentiviruses with control and H3L^OE^ were intraperitoneally injected into one month old mouse. Two months later, heart functions were evaluated. IL1A blocking antibody was also injected to evaluate whether IL1A blockage can attenuate in vivo heart injuries induced by H3L^OE^. **B** The enzyme-linked immunosorbent assay (ELISA) showing IL1A protein expression level in blood plasma from mouse heart. *p < 0.05. Expression level in the Y-axis meant the read count on the absorption at 450 nm by the equipment. **C** Echocardiography showing B-Mode image of the short axis view of left ventricle of mice hearts. Echocardiography was performed on the Vevo 2100 Imaging System (Visualsonics) on conscious mice. **D** Echocardiography showing ejection fraction (EF, %). Echocardiography was performed on conscious mice. *p < 0.05. **E** Echocardiography showing fractional shortening (FS, %). Echocardiography was performed on conscious mice. *p < 0.05. **F** Echocardiography showing Left ventricular posterior wall end diastole (LVPW-d, mm). Echocardiography was performed on conscious mice. *p < 0.05. **G** Hematoxylin and Eosin (HE) staining of mouse heart sections embedded with paraffin. Scale bar, 1 mm. **H** WGA staining of mouse heart sections. Green color showed WGA signal. Blue color showed DAPI (nucleus). Scale bar, 100 µm. *p < 0.05. **I** Masson staining of mouse heart transverse sections. Red color showed muscle tissues. Blue color showed collagens. Scale bar, 100 µm. **J** Masson staining of mouse heart longitudinal sections. Scale bar, 100 µm. Red color showed muscle tissues. Blue color showed collagens. Scale bar, 50 µm. **K** The statistics data of collagen percentage in heart sections from (**J**). *p < 0.05. **L** Western blot showing the protein expression of COL1A1 and NPPB in mouse heart tissues. *p < 0.05. **M** Western blot showing the protein expression of COL3A1 in mouse heart tissues. *p < 0.05.

Having established that H3L can induce transcriptional perturbations in mouse heart, we proceeded to assess whether it could lead to cardiac dysfunctions in vivo (Fig. 6A). Moreover, considering our previous findings suggesting that targeting IL1A might hold promise as a potential therapeutic strategy (Fig. 2M, N), we aimed to evaluate whether blocking IL1A could attenuate the in vivo injuries caused by H3L (Fig. 6A). To investigate this, we administered H3L lentiviruses, with or without IL1A blocking antibody, to the mice (Fig. 6A). After two months, we found that IL1A in mouse blood was significantly up-regulated by the overexpression of H3L (Fig. 6B), which was similar with the phenotype of IL1A upregulation in human cells in vitro (Fig. 2). Echocardiography results (Fig. 6C) revealed that H3L^OE^ mice exhibited significantly lower Ejection Fraction (EF, %) (Fig. 6D) and Fractional Shortening (FS, %) (Fig. 6E) compared to Control mice. Nevertheless, antibody blocking IL1A mitigated the phenotypes of lower EF and FS induced by H3L^OE^ (Fig. 6C–E). Additionally, we uncovered that LVPW-d (left ventricular posterior wall thickness in diastole, mm) was increased in H3L^OE^ mice (Fig. 6F). However, when IL1A-blocking antibody was applied, this cardiac injury was significantly mitigated (Fig. 6F). Histochemistry on heart sections showed that H3L^OE^ induced cardiac hypertrophy, which was also attenuated by IL1A-blocking antibody (Fig. 6G, H). Furthermore, we noted that H3L^OE^ slightly but significantly induced cardiac fibrosis in the heart tissues, and IL1A-blocking antibody mitigated this phenotype as well (Fig. 6I–K). At protein level, H3L overexpression upregulated expression levels of cardiac hypertrophy marker (NPPB) (Fig. 6L) and cardiac fibrosis markers (COL1A1 and COL3A1) (Fig. 6L, M, Fig. S7E). The data demonstrated that H3L induces in vivo cardiac remodeling.

### H3L induces injuries in mouse cardiomyocytes, which are attenuated by repressing IL1A

**Fig. 7:**
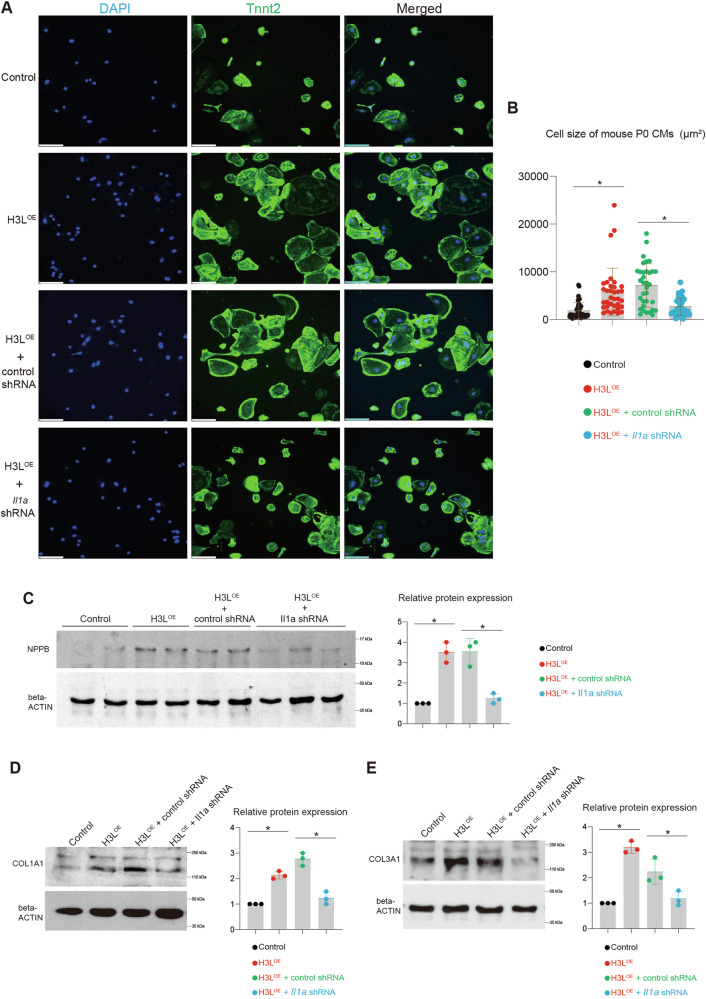
H3L induces injuries in mouse cardiomyocytes in vitro, which are attenuated by repressing IL1A. **A** Immunostaining showing mouse P0 neonatal cardiomyocytes. TNNT2 is a specific marker of cardiomyocyte. Scale bar, 100 µm. **B** Statistic analysis of cell size of mouse P0 cardiomyocytes from (**A**). *p < 0.05. **C** Western blot showing the protein expression of NPPB in mouse cardiomyocytes. *p < 0.05. **D** Western blot showing the protein expression of COL1A1 in mouse cardiomyocytes. *p < 0.05. **E** Western blot showing the protein expression of COL3A1 in mouse cardiomyocytes. *p < 0.05.

To further corroborate the roles of H3L and IL1A in mouse heart, we conducted an assessment of their functions in mouse neonatal (P0) cardiomyocytes by knocking down *Il1a*. Our data revealed that overexpression of H3L prompted cardiomyocyte hypertrophy (Fig. 7A, B). When *Il1a* was suppressed using shRNA, it resulted in a significant reduction in H3L-induced cardiomyocyte hypertrophy (Fig. 7A, B). Furthermore, at the protein level, the overexpression of H3L substantially increased the expression levels of cardiac hypertrophy markers (NPPB) (Fig. 7C) and cardiac fibrosis markers (COL1A1 and COL3A1) (Fig. 7D, E, Fig. S7F–H). Conversely, the knockdown of *Il1a* through shRNA mitigated the H3L-induced upregulation of these cardiac remodeling markers (Fig. 7C–E, Fig. S7F–H). This finding demonstrated that H3L induces injuries in mouse cardiomyocytes, which can be attenuated by repressing IL1A.

## Discussion

Until now, whether and how the MPV components directly cause injuries in human are still unclear. In this study, we established a platform to screen which MPV proteins could cause injuries in human and mouse. The findings provided a comprehensive understanding of the functional roles of the MPV protein H3L, in human and mouse, with a specific focus on its impact in the cardiac system. The results highlighted the potential mechanisms by which H3L induce expression perturbations, DNA damage and cardiac injuries, shedding light on the complex interplay between the virus component and host cells. Our findings would provide promises for therapies of MPV-associated diseases.

There may be a potential difference between artificially induced overexpression of the H3L gene and its expression during real MPV infection. We propose that during a real MPV infection, H3L overexpression should occur as the virus replicates and spreads in animal and human cells, leading to an increase in viral protein expression [37]. When a virus infects a host cell, it hijacks the cellular machinery to replicate itself, leading to the production of viral proteins [37]. In the case of MPV, its replication cycle would likely involve overexpression of various viral proteins, including H3L, as part of its strategy to propagate within the host. This rationale underscores that artificially inducing overexpression of H3L may be a viable approach to studying its function. Artificial overexpression mimics the conditions of MPV infection-induced H3L overexpression, facilitating the exploration of H3L’s role. Experimental investigations could involve infecting cells with MPV and tracking H3L expression levels over time to determine if they correlate with viral replication dynamics or match levels seen in artificial overexpression studies. However, it is important to note that other MPV viral proteins may also have pathogenic functions, complicating the isolation of the specific phenotype caused by individual H3L.

One of our crucial observations showed that H3L led to expression perturbations, particularly up-regulating genes in cell death and DNA damage. These findings are of great significance, as they provide insights into the molecular mechanisms by which MPV infection may cause tissue damage and potentially contribute to disease progression. The activation of genes involved in apoptosis and DNA damage signaling pathways suggests that H3L may directly influence cellular processes that lead to cell death and genomic instability. Cell death is a well-known outcome of virus infection, and studies have highlighted the critical roles played by both the core machinery and specific viral components in inducing this process [38]. Viruses often manipulate host cellular processes by targeting and recruiting host proteins to facilitate their replication [39]. Through direct interactions and hijacking of human proteins, viral proteins can disrupt normal physiological functions in the host, resulting in cellular damages [39, 40]. Therefore, studying the specific functions of viral components in host human cells is vital for gaining a deeper understanding of viral pathogenesis. MPV has already caused significant outbreaks globally, and remains a risk to human health. However, there have been limited studies focused on understanding whether and how MPV induces injuries in human cells. This knowledge gap has greatly hindered the development of specific drugs or vaccines to target MPV or to treat MPV-associated injuries. In our study, we hypothesized that MPV-induced damages in human cells could be attributed to specific viral proteins, as is often the case with viruses that induce injuries through their components, such as membrane proteins, DNA-binding proteins, and RNA-binding proteins [38]. To shed light on MPV pathogenesis, we initially screened four of the core MPV proteins and found that H3L directly induced DNA damage. This suggests that H3L is a key contributor to MPV-induced injuries in human cells. Notably, H3L is surface membrane protein, which is often exposed to the host’s immune system during infection. This highlights its potential as target for the design of specific vaccines or drugs to combat MPV-induced injuries.

The activation of Interferon and Interleukin-4/Interleukin-13 signaling pathways by H3L also deserves further attention. These pathways are key components of the host immune response to viral infections [41]. The upregulation of genes involved in these signaling pathways implies that H3L might be manipulating the host immune response to facilitate viral replication or evade the immune system. This finding raises intriguing questions about the interaction between the MPV and the human immune system and the potential implications for antiviral strategies. The identification of IL1A as a downstream effector of H3L in our study is a significant observation in understanding the molecular mechanisms underlying MPV-induced pathogenesis. IL-1A is expressed in many tissues and by diverse cell types, such as fibroblasts, hepatocytes, keratinocytes, macrophages, dendritic cells and T cells [42, 43]. IL1A is a pro-inflammatory cytokine known to be involved in various cellular processes, including inflammation, cell death, and tissue repair [29, 44, 45]. Inflammatory cytokines associated with damaging responses are often members of the IL-1 family, such as IL-1A and IL-1B [46]. IL-1A promotes inflammation, apoptosis, necrosis and tissue damage, finally resulting in cell death [29, 43–45]. IL1A blocking or IL1A receptor antagonist may protect against a broad spectrum of diseases [43, 47, 48]. However, whether and how IL1A are involved in MPV-induced injuries in human are unclear. This study demonstrated that H3L drives the upregulation of IL1A through direct binding to IL1A promoter to increase its expression, or promoting the expression of transcription factor IRF4, which, in turn, upregulated IL1A via binding to IL1A promoter. Subsequently, IL1A was shown to induce DNA damage in human cells, which could be mitigated by IL1A-blocking antibody in our study. This finding firstly establishes a link between MPV viral protein and the cellular machinery that leads to injuries in human, and also highlights the potential therapeutic implications of targeting IL1A to mitigate MPV-induced injuries.

The impact of H3L on cardiac system is a particularly intriguing aspect in our study, since we discovered that H3L overexpression perturbated transcriptions in human cardiac genes. The further observation that it not only inhibits mesoderm differentiation and cardiomyocyte specification but also promotes DNA damage raises questions about its potential contribution to cardiac pathologies. Although cardiac complications, such as pericarditis and myocarditis, were reported to be associated with MPV infection in some patients [2–7], whether MPV infection is the causative for cardiac complications and what viral protein directly cause cardiac injuries are fully unclear. In this study, we found that H3L overexpression perturbated transcriptions in cardiac genes and induced DNA damage in human cells, including hESCs, hESC-derived cardiac lineage cells and hESC-derived cardiomyocytes. Furthermore, in this study, the perturbated transcriptions in mouse heart tissues, leading to the induction of cardiac injuries in mouse upon overexpression of H3L, further supports their causative roles in cardiac damages. This also may explain why the cardiac complications could be observed in MPV-infected patients [2–7]. We also observed that IL1A expression was also activated in mouse by H3L, which phenocopied the results in human cells. IL1A is detectable in mouse cardiomyocytes and induce inflammation in heart after acute myocardial infarction (AMI), which can be mitigated by Il1a knockout [49]. Inhibition of interleukin-1 by anakinra or IL1 receptor antagonist, which blocks IL1A and IL1B, protect against cardiac injuries and improve vascular and left ventricular function [50–53]. These evidences show that IL1A is a potential target for cardiac inflammation treatment. However, although IL1A is activated by H3L, whether blocking IL1A can attenuate H3L-induced cardiac injuries is unclear. Our further finding demonstrated that IL1A blockade can attenuate these cardiac injuries. Thus, our finding firstly underscores the importance of IL1A as a potential therapeutic target for cardiac complications associated with MPV infection.

In the reports, some patients infected by MPV had acute cardiac injuries such as pericarditis and myocarditis [3, 4, 7, 54], some of which had ST-elevation and sinus tachycardia observed in electrocardiogram [3]. Acute cardiac injuries may cause sudden death. It is possible that MPV infection also have delayed or long-term effects on human heart such as inducing cardiomyopathy and ventricular arrhythmias [3], although they remain to be further confirmed and studied. In this study, we demonstrated that MPV protein H3L directly induced transcriptional perturbations in adult mouse heart, leading to cardiac hypertrophy, fibrosis and cardiac dysfunction, some of which could be observed in neonatal cardiomyocytes derived from mouse. Our findings provided evidence that we cannot ruled out the possibility of MPV as a new emerging cardiotropic virus and the possibility that cardiac complications will be a new threat for human. Consequently, it remains to be necessary and urging to develop a deeper understanding of MPV and MPV-associated cardiac injuries [5].

One of the important findings in our study was the H3L-induced transcriptional perturbations in genes involved in epigenetic regulation. We discovered that Histone 3 protein level was significantly changed. It was reported that MPV infection induced expression change of histone at RNA level in monkey cells [55]. This indicated that MPV infection might affect chromatin status. However, whether MPV remodels chromatin and what viral protein is involved in chromatin re-modeling are still unclear. In this study, we found that a small part of H3L protein was localized in nucleus. We posited that H3L localized in nucleus could remodel chromatin in inducing injuries, because in the initial stages of infection, host chromatin proteins can be mobilized and recruited to viral genomes, resulting in dramatic damages in cells [56, 57]. As the infection proceeds, cellular chromatin must be structurally re-organized to make room for viral replication compartments that eventually coalesce and fill much of the nucleus [58, 59]. Viruses often inhibit or exploit specific cellular proteins to promote viral replication and facilitate maintenance of the viral genome. The repertoire of host proteins targeted directly or indirectly by viruses has been expanded to include histones [60]. This section will consider several examples of specific viral proteins being necessary and sufficient to induce or remove post-translational modifications on histones [61]. Nevertheless, whether H3L can remodel chromatin is fully unknown and remains to be elucidated. Our findings demonstrated that H3L directly occupy promoters of genes, leading to pattern changes in lysine methylation of histone 3. Thus, the final observations are that H3L represses expressions of cardiac genes but promote expressions of IL1A and P53, which cause DNA damage/cell death and cardiac dysfunctions. We here provide a mechanistic link between the MPV viral protein, chromatin remodeling, epigenetic regulation and the modulation of gene expression, further emphasizing its role in expression perturbations.

## Conclusions

Overall, our study provides a comprehensive understanding of the functional roles of a MPV protein (H3L) in human and mouse, and its impact on cardiac system. The findings underscore the complexity of the interactions between MPV and the host, and highlight potential targets for therapeutic interventions. The research also opens up exciting avenues for future investigations into the broader implications of MPV viral proteins in viral pathogenesis and host responses to MPV infection.

## Materials and methods

### Human embryonic stem cells and cardiac development model

Human embryonic stem cells (hESCs) were routinely maintained in mTesR1 medium (STEMCELL Technologies). HESCs, which were differentiated towards mesodermal cells, cardiac progenitor cells and cardiomyocytes, were used as cardiac development model [62–64]. Cardiac differentiation was induced by STEMdiff™ Ventricular Cardiomyocyte Differentiation Kit (STEMCELL Technologies) according to the manual.

### Monkeypox virus protein and DNA sequences

Monkeypox virus protein sequences were from NCBI database (https://www.ncbi.nlm.nih.gov/nuccore/ON563414). Monkeypox virus was isolated from patient MPXV_USA_2022_MA001, complete genome. Please see detailed information in the Supplemental information.

### Mouse model

The protocols used in this study were approved by the Institutional Review Board (IRB) at Guangdong Provincial People’s Hospital and Guangdong Academy of Medical Sciences (Guangzhou, China). And all animal experiments were conducted in accordance with the Institutional Review Board (IRB) at Guangdong Provincial People’s Hospital and Guangdong Academy of Medical Sciences (Guangzhou, China). The C57BL/6J mice, purchased from Cyagen Biosciences (Guangzhou, CHINA), were used throughout this study. One month old mice were intraperitoneally injected with the lentivirus (0.1 × 10^9^ TU per mouse). After two months post injection, the mice were used for echocardiography and other experiments. High-titer stock (1 × 10^9^ transducing units [TU]/mL) of a second-generation lentivirus in which expression of gene is driven EF-1α promoter with myc tag on N terminal was used in this study for mouse model. All mice were housed under specific pathogen-free (SPF) conditions with standard chow and bedding with 12 h day/night cycle according to institutional protocols. Animals of male were applied in this study.

### Mouse Echocardiography and Electrocardiogram (ECG)

Echocardiography was performed using the Vevo 2100 ultrasound system (VisualSonics, Canada) equipped with a MS-550 linear-array probe working at a central frequency of 40 MHz. After the animals were anesthetized with 3.0% (v/v) isoflurane carried by pure oxygen, they were placed at supine position on a pre-warmed platform at around 37 °C. Then, hair removal cream was used to remove hair on chest and abdomen. Subsequently, the anesthesia was not maintained and echocardiography was performed under conscious condition. The eye gel was used to prevent ocular dehydration. Needle probes attached to ECG leads embedded in the imaging platform were subcutaneously inserted to each limb for ECG. ECG was monitored and maintained during the whole echocardiography procedure. Left ventricular (LV) geometry and function were evaluated using M-mode from parasternal short-axis. LV anterior (LVAW) and posterior (LVPW) wall thickness and internal dimensions (LVID) were evaluated at the M-mode during systole (s) and diastole (d). LV ejection fraction (EF) was calculated from the volumes (Vol), which are computed according to the Teichholz formula. And the fractional shortening (FS) was also calculated. Data was transferred to an offline computer and analyzed with Vevo 2100 software (VisualSonics, Canada) by a technician blinded to the study groups. ECG data were recorded and analyzed using the MedLab-U/4C501H equipped with the ECG Analysis Module (SHENJIAN company, Shanghai, China). Peak amplitudes and intervals of ECG were determined by the equipment. After echocardiography and ECG, mice were euthanized via cervical dislocation under anesthesia and hearts were dissected for other experiments.

### RNA-seq

Total RNAs were purified with RNeasy Kit (Qiagen) and miRNeasy Kits (Qiagen). mRNAs were purified from the total RNA using magnetic beads attached to poly-T oligos. First strand cDNA was synthesized using random hexamer primers and M-MuLV Reverse Transcriptase, followed by degradation of RNA using RNaseH. Second strand cDNA synthesis was performed with DNA Polymerase I and dNTPs. cDNA fragments of preferred length (370–420 bp) were purified using the AMPure XP system. The library was then subjected to PCR amplification, purified with AMPure XP beads, and the final library was obtained. After library qualification, different libraries were pooled based on their effective concentration and the desired amount of data, and then sequenced using the Illumina NovaSeq 6000. FeatureCounts (v1.5.0-p3) was used to count the reads numbers mapped to each gene. Differential expression analysis was performed using the DESeq2 R package (1.20.0). The resulting *P* values were adjusted using the Benjamini and Hochberg’s approach for controlling the false discovery rate. P < 0.05 and |log_2_(fold change) | > 0 were set as the threshold for significantly differential expression.

For enrichment analyses of significantly differential genes, Gene Ontology (GO), KEGG, and GSEA were independently performed. GO enrichment analysis of differentially expressed genes was implemented by the clusterProfiler R package (3.8.1) and THE GENE ONTOLOGY RESOURCE (http://geneontology.org/). ClusterProfiler R package (3.8.1) was used to test the statistical enrichment of differential expression genes in KEGG pathways. Local version of the GSEA analysis tool (http://www.broadinstitute.org/gsea/index.jsp) was used. All terms with corrected P value less than 0.05 were considered significantly enriched by differential expressed genes. The RNA-seq and data analysis were conducted in Novogene Corporation (Beijing, China).

### Chromatin immunoprecipitation followed with sequencing (ChIP-seq) and ChIP-qPCR

HESCs were cultured in mTesR1 medium in P10 plate. For chromatin shearing, truChIP® Chromatin Shearing Kit (Covaris, USA) was used according to the manual. Briefly, when cells density as around 100%, all cells were crosslinked and lysed by truChIP® Chromatin Shearing Kit (Covaris, USA). Then nuclei were isolated and sheared by truChIP® Chromatin Shearing Kit (Covaris, USA). The isolated chromatins were sheared on ME220 Focused-ultrasonicator (Covaris, USA). The shearing program was: Min/Max Temperature, 6-12 degree; Target Size (bp), 200 to 500; PIP, 75; Duty Factor (%), 15; CPB, 1000; shearing Time (minutes), 20 min; Water Level, 9; Sample Volume (ml), 1 ml. For ChIP, EZ-Magna ChIP™ A/G Chromatin Immunoprecipitation Kit (Millipore, USA) was used according to the manual. The purified DNA was used for ChIP-qPCR. All ChIP–qPCR data presented were at least three biological replicates. Primer sequences were shown in Table S1. Oligonucleotides.

The purified DNA was also used for ChIP-seq library preparation. Subsequently, pair-end sequencing of sample was performed on NovaSeq 6000 (Illumina, USA). Raw data (raw reads) of fastq format were firstly processed using fastp software (version 0.19.11). Clean data (clean reads) were obtained for next analysis. Index of the reference genome was built using BWA (v 0.7.12) and clean reads were aligned to the reference genome using BWA mem (v 0.7.12). After mapping reads to the reference genome, MACS2 (version 2.1.0) was used for peak calling to identify regions of IP enrichment over background. A q-value threshold of 0.05 was used for all data sets. After peak calling, the distribution of chromosome distribution, peak width, fold enrichment, significant level and peak summit number per peak were all displayed. ChIPseeker was used to retrieve the nearest genes around the peak and annotate genomic region of the peak. Different peak analysis was based on the fold enrichment of peaks of different experiments. A peak was determined as different peak when the odds ratio between two groups was more than 2. Genes associated with different peaks were identified for Gene Ontology (GO) and KEGG enrichment analysis. GO enrichment analysis was implemented by the GOseq R package, in which gene length bias was corrected. GO terms with corrected P value less than 0.05 were considered significantly enriched by peak-related genes. KEGG was analyzed and KOBAS software was used to test the statistical enrichment of peak related genes in KEGG pathways. DNA sequencing and data analysis were constructed by Novogene Corporation (Beijing, China).

### The enzyme-linked immunosorbent assay (ELISA)

For the ELISA of hESCs cultured in mTesR1 medium, Human IL-1A ELISA Kit (Invitroge, BMS243-2) was used. Briefly, the medium was daily replaced with fresh mTesR1 medium. One next day, the supernatant medium and the cells were collected for ELISA experiment, separately. ELISA of the supernatant medium was performed according to the manual without dilution. For cells, they were lysed with cOmplete lysis buffer (Roche, USA) supplemented with protease inhibitor cocktail (cOmplete) (Roche, USA) according to the manual. Then, isolated protein lysis with 1:200 dilution was used for ELISA according to the manual. The signals were captured by Luminescence Microplate Readers. For the ELISA of mouse blood, plasma and cells of 50 µl blood were isolated by centrifuge. Plasma with 1:2 dilution was used for ELISA by using Mouse IL-1 alpha ELISA (RayBiotech, ELM-IL1a). Cells were lysed with cOmplete lysis buffer (Roche, USA) supplemented with protease inhibitor cocktail (cOmplete) (Roche, USA) according to the manual. Then, the cell lysis with 1:2 dilution was used for ELISA. The signals were captured by Luminescence Microplate Readers.

### Western blot

Western blot analysis was performed using standard procedures. Cells were lysed with cOmplete lysis buffer (Roche, USA) supplemented with protease inhibitor cocktail (cOmplete) (Roche, USA) according to the manual. The concentration of isolated proteins was determined with a BCA protein assay kit (Thermo Fisher Scientific, USA) according to the manufacturer’s instructions. After lysates were denaturized for 5 min at 95 °C on heater, electrophoresis and transfer blotting were performed on the Trans-Blot Turbo system (Bio-Rad, USA) according to the manufacturer’s manuals. Membranes were blocked with 10% milk in 1× TBST (0.05% Tween-20 in 1× TBS buffer) at room temperature for 1 h and then incubated with the 1st antibody (diluted in 1× TBST) at 4 °C overnight. On next day, membranes were incubated with the 2nd antibody at room temperature for 1 h. Membranes were washed and then visualized with Clarity Western ECL Substrate (Bio-rad, USA). The images were captured with the ImageQuant LAS 500 system (GE HealthCare, USA).

### RT-qPCR

Total RNAs were isolated by miRNeasy mini kit (Qiagen, USA) or RNeasy Mini Kit (Qiagen, USA) according to the manuals. Quantity and quality of total RNAs was determined by Nanodrop 2000 Spectrophotometer (Thermo Fisher, USA). Fresh total RNAs were used for RNA-seq or RT-qPCR. Up to 1 µg of isolated RNA was used for reverse transcription with 1st strand cDNA Synthesis Kit (Takara Bio, Japan). Real-time qPCR was performed by using SYBR Premix Ex Taq (Takara Bio, Japan) in a Biorad Real-Time PCR System (Biorad, USA) according to the manufacturer’s instructions. All PCR reactions were performed in at least three biological triplicates, normalized to the internal control genes GAPDH or beta-actin, and analyzed by using the comparative 2^-ΔΔCt^ method. All primer sequences were listed in Table S1. Oligonucleotides.

### Flow cytometry

Cultured cells were fixed by 4% PFA for 10 min at room temperature. Cells were then incubated with 1st antibody in blocking buffer (10% BSA and 0.1% saponin plus 0.1% Triton-100 in 1× PBS) at 37 °C for 1 h. Cells were then washed with 1× PBS for three times, following with incubation with 2nd antibody in blocking buffer (containing 10% BSA and 0.1% saponin plus 0.1% Triton-100 in 1× PBS) at 37 °C for 1 h. For terminal deoxynucleotidyl transferase biotin-dUTP nick end labeling (TUNEL), the labeling buffer containing enzyme was added and mixed together with secondary antibody according to TUNEL kit (Sigma-Aldrich) manual. Finally, cells were washed three times with 1× PBS buffer. Flow cytometry evaluation was performed on CytoFLEX Flow Cytometer (Beckman Coulter, USA). Data were analyzed by FlowJo (Treestar, USA).

### Immunostaining

Cultured cells on slides were fixed with 4% paraformaldehyde (PFA) for 10 min at room temperature. Then cells were blocked with 10% bovine serum albumin (BSA) (STEM CELL Technologies) in block buffer (0.1% saponin plus 0.1% Triton-100 in 1× PBS) for 1 h, followed by overnight incubation with primary antibody at 4 °C. On next day, cells were washed three times with 1× PBS buffer, and then incubated for 1 h at room temperature in the dark room with secondary antibody in block buffer (0.1% saponin plus 0.1% Triton-100 in 1× PBS). For the experiment of terminal deoxynucleotidyl transferase biotin-dUTP nick end labeling (TUNEL) (Sigma-Aldrich), the labeling buffer containing enzyme was added and mixed together with secondary antibody according to the manual. Finally, cells were washed again three times with 1× PBS, and then mounted with DAPI solution. Images were captured with the Zeiss ZEN confocal microscope equipped with an oil immersion objective.

### Histological staining

Mice hearts were freshly harvested, fixed in 10% neutral buffered formalin, and processed for paraffin embedding. Paraffin-embedded hearts were sectioned at a thickness of 6 µm and mounted on positively charged slides. H/E staining was performed by using H&E Staining Kit (Hematoxylin and Eosin) (Abcam, ab245880) according to the manual. Masson staining was performed by using Epredia™ Richard-Allan Scientific™ Masson Trichrome Kit (Fisher Scientific, 22-110-648) according to the manual. Sections were scanned by using slide scanning image system SQS-120P-20 (Shengqiang Technology, China).

### Quantification and statistical analysis

Data were represented as mean ± SD of biological replicates. Individual data points of sample number were also shown. Statistical significance was evaluated by using unpaired Student’s *t* test (two-tailed) (comparison between two groups). One-Way ANOVA was used to compare more than two groups. P value less than 0.05 was considered statistically significant.

### Supplementary information

supplemental figure 1

supplemental figure 2

supplemental figure 3

supplemental figure 4

supplemental figure 5

supplemental figure 6

supplemental figure 7

supplemental figure legends

Supplementary Information

Table S1. Oligonucleotides

Table S2. gene_readcount_RNA-seq for Figure 1

Table S3. gene_readcount_RNA-seq for Figure 3

Table S4. gene_readcount_RNA-seq for Figure 5

Table S5. peaks_ChIP-seq for Figure 4

Supplemental material-Western blots

## Data Availability

All sequencing data were deposited to NCBI GSE database. Accession numbers of RNA-seq are GSE235128, GSE239712 and GSE240139. Accession number of ChIP-seq is GSE239888.
