# Supplementary figures and images for: Monkeypox virus protein H3L induces injuries in human and mouse
Source: Cell Death Dis. 2024 Aug 21;15(8):607. doi: 10.1038/s41419-024-06990-2 (PMC11339448; doi:10.1038/s41419-024-06990-2)

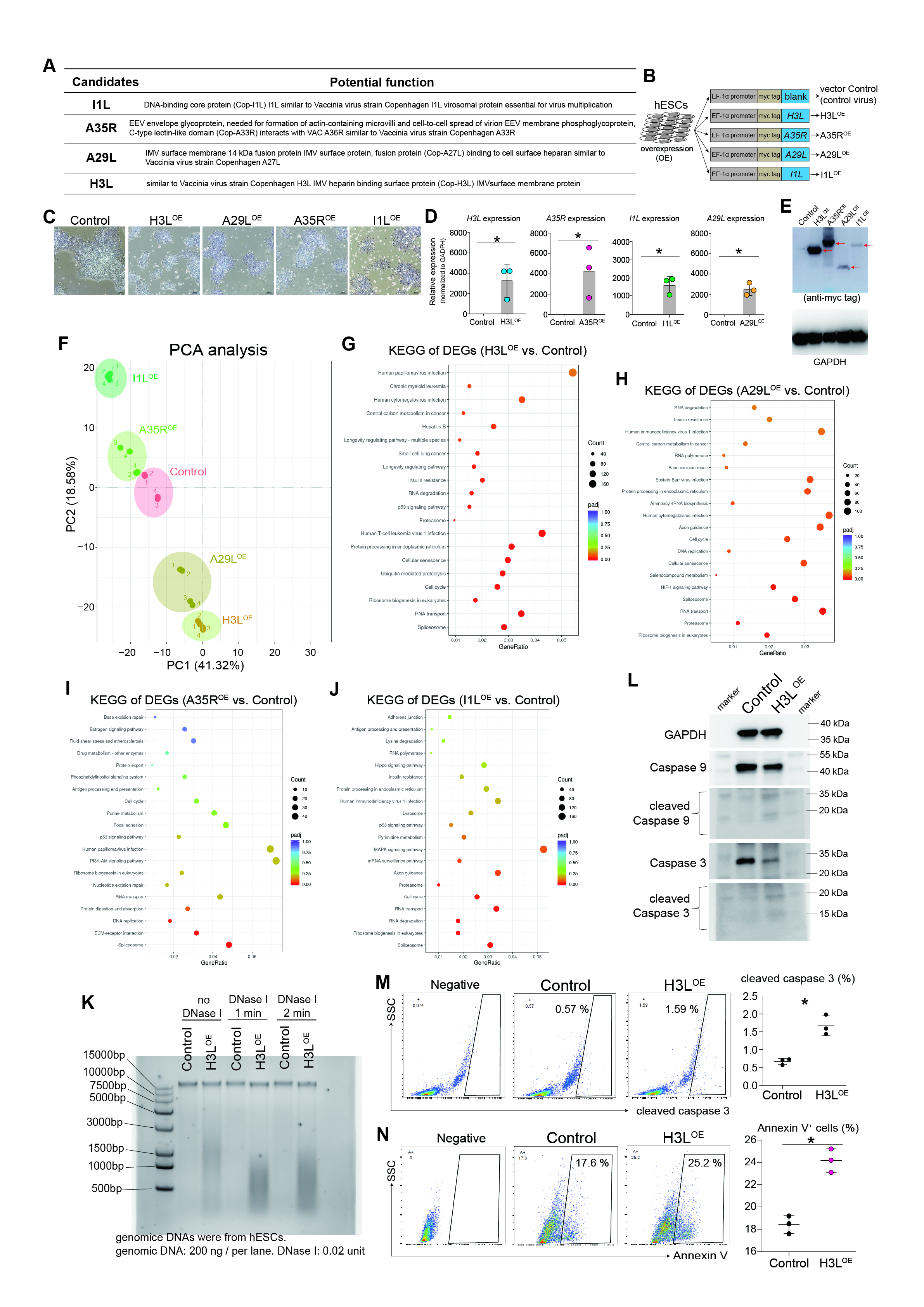

Supplement: Supplementary file 1 — supplemental figure 1 [file 41419_2024_6990_MOESM1_ESM.jpg]

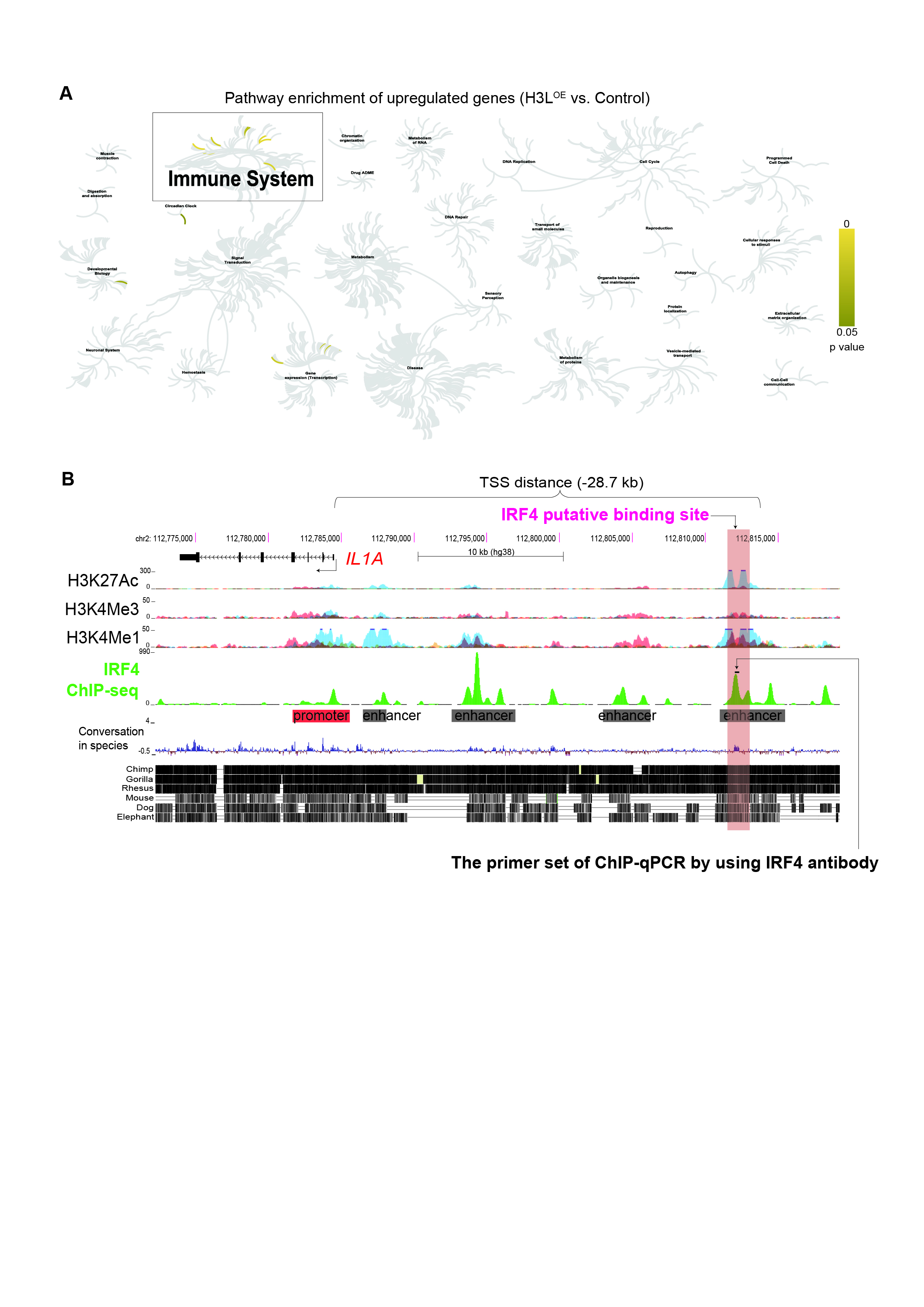

Supplement: Supplementary file 2 — supplemental figure 2 [file 41419_2024_6990_MOESM2_ESM.jpg]

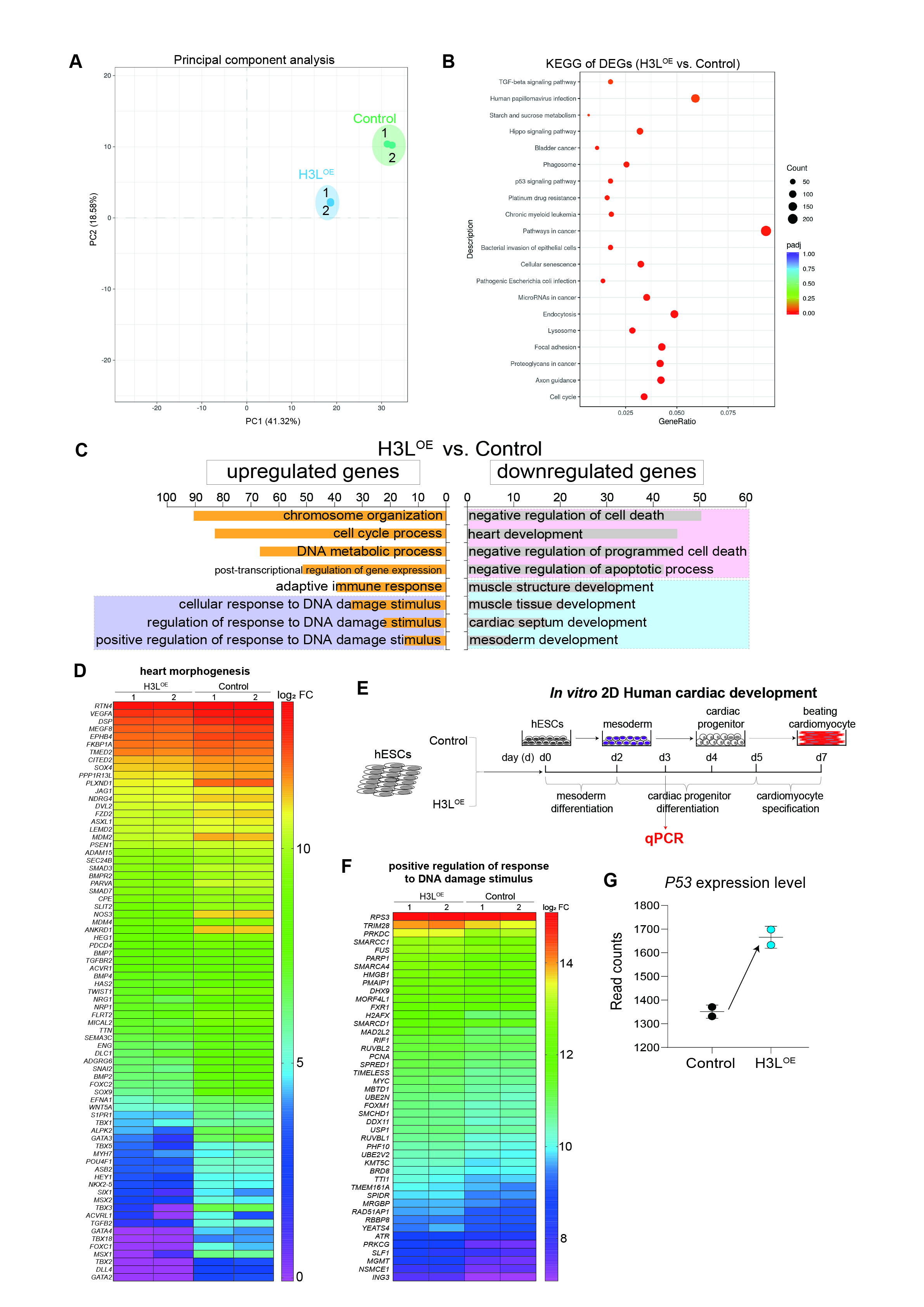

Supplement: Supplementary file 3 — supplemental figure 3 [file 41419_2024_6990_MOESM3_ESM.jpg]

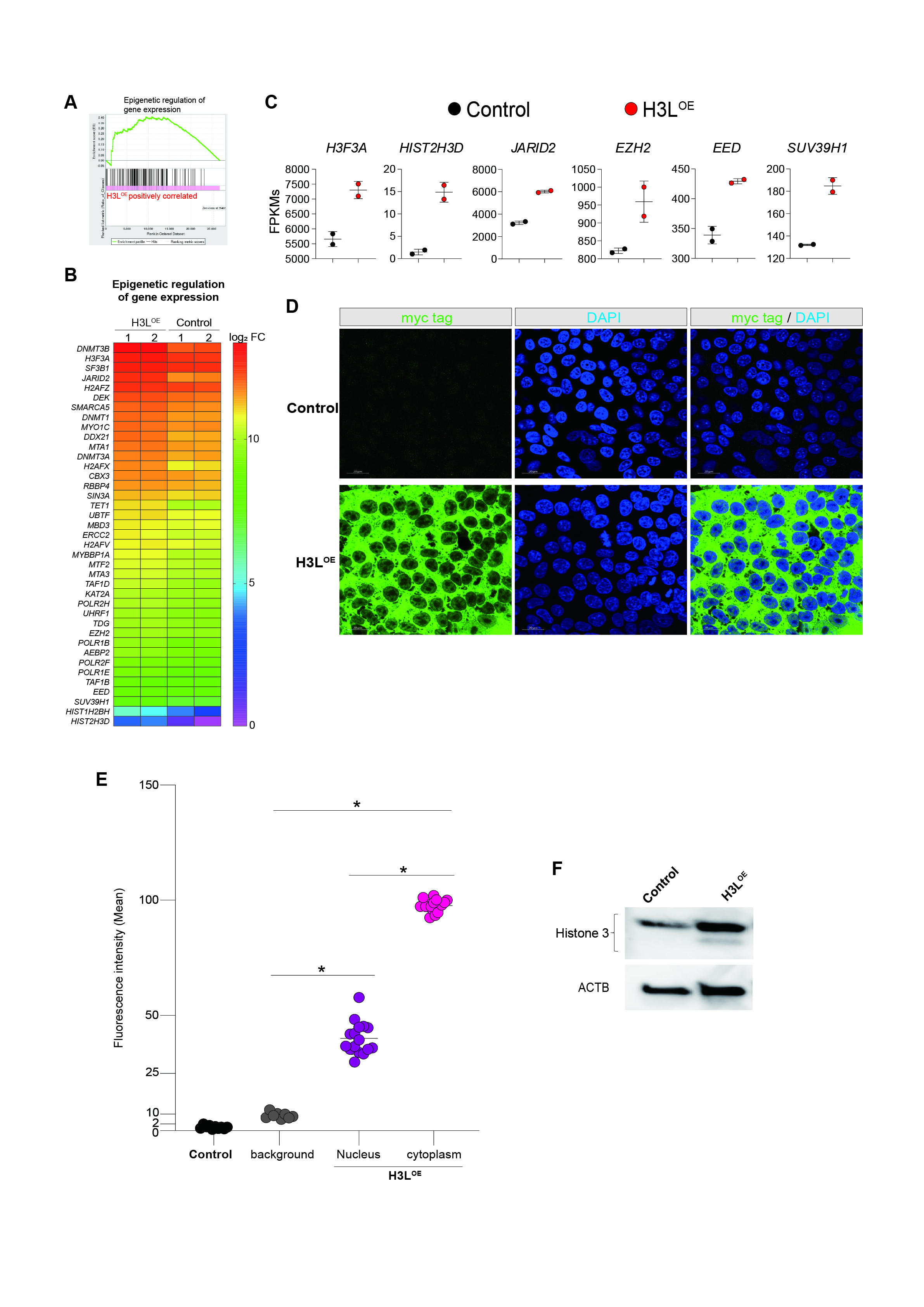

Supplement: Supplementary file 4 — supplemental figure 4 [file 41419_2024_6990_MOESM4_ESM.jpg]

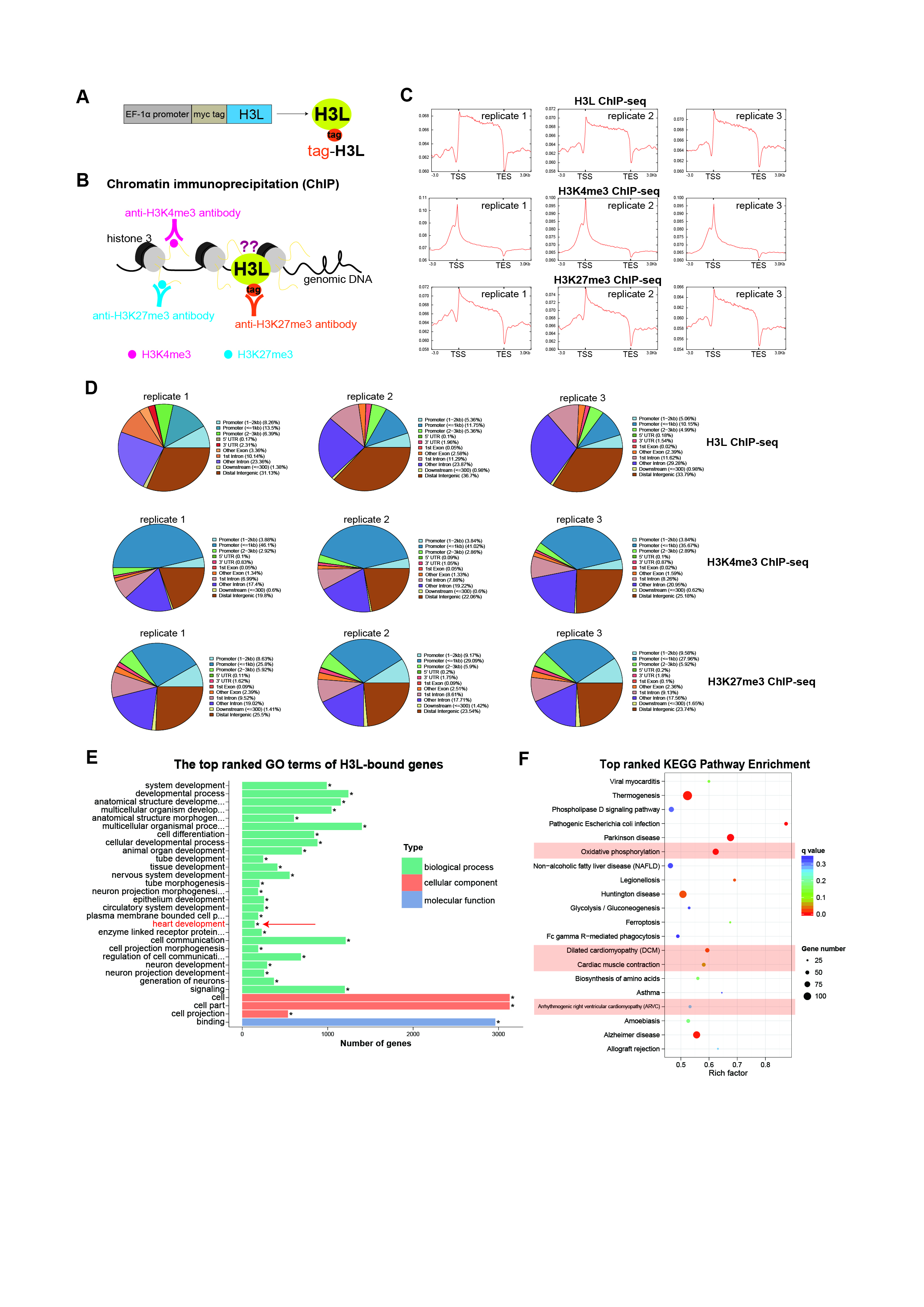

Supplement: Supplementary file 5 — supplemental figure 5 [file 41419_2024_6990_MOESM5_ESM.jpg]

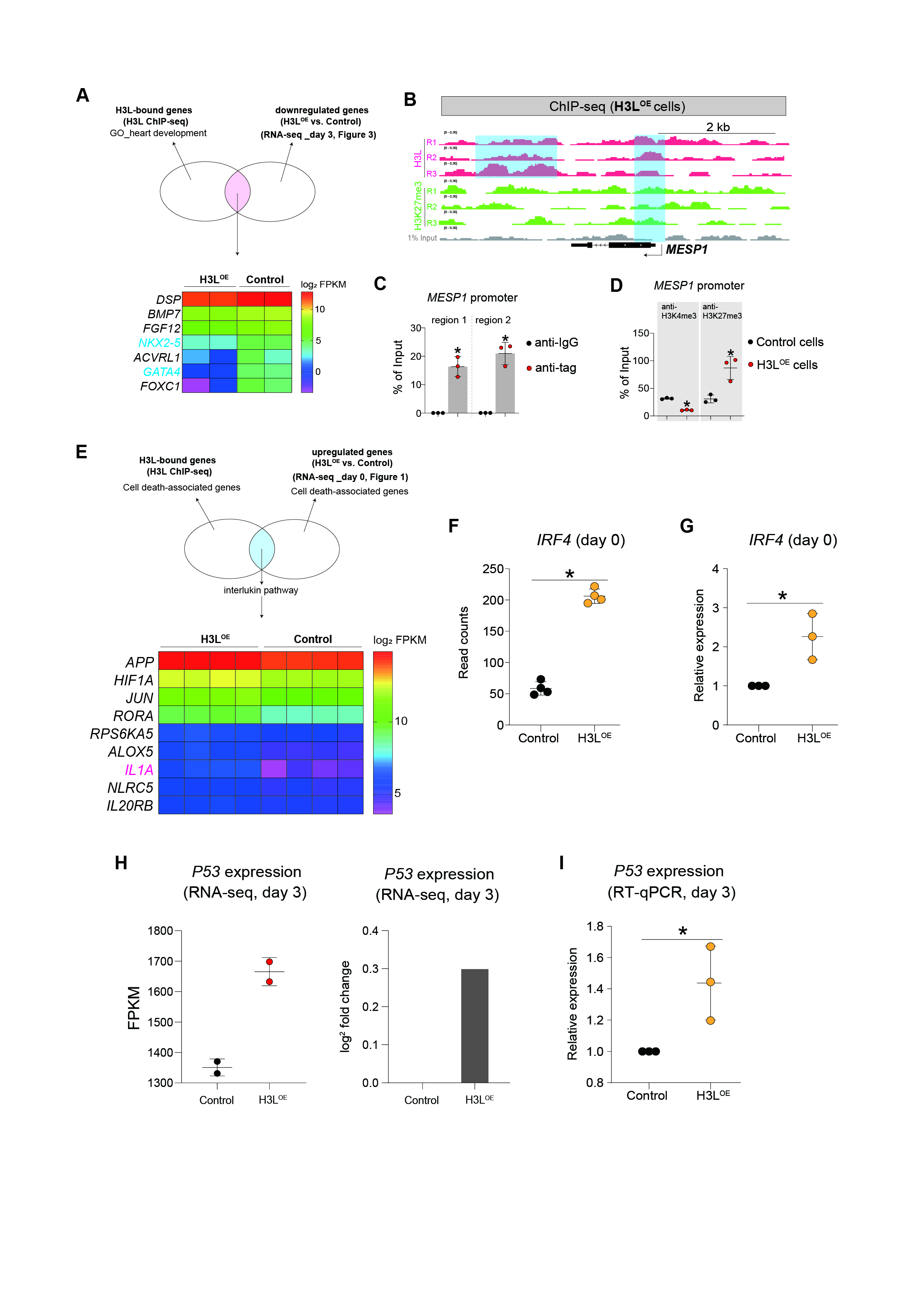

Supplement: Supplementary file 6 — supplemental figure 6 [file 41419_2024_6990_MOESM6_ESM.jpg]

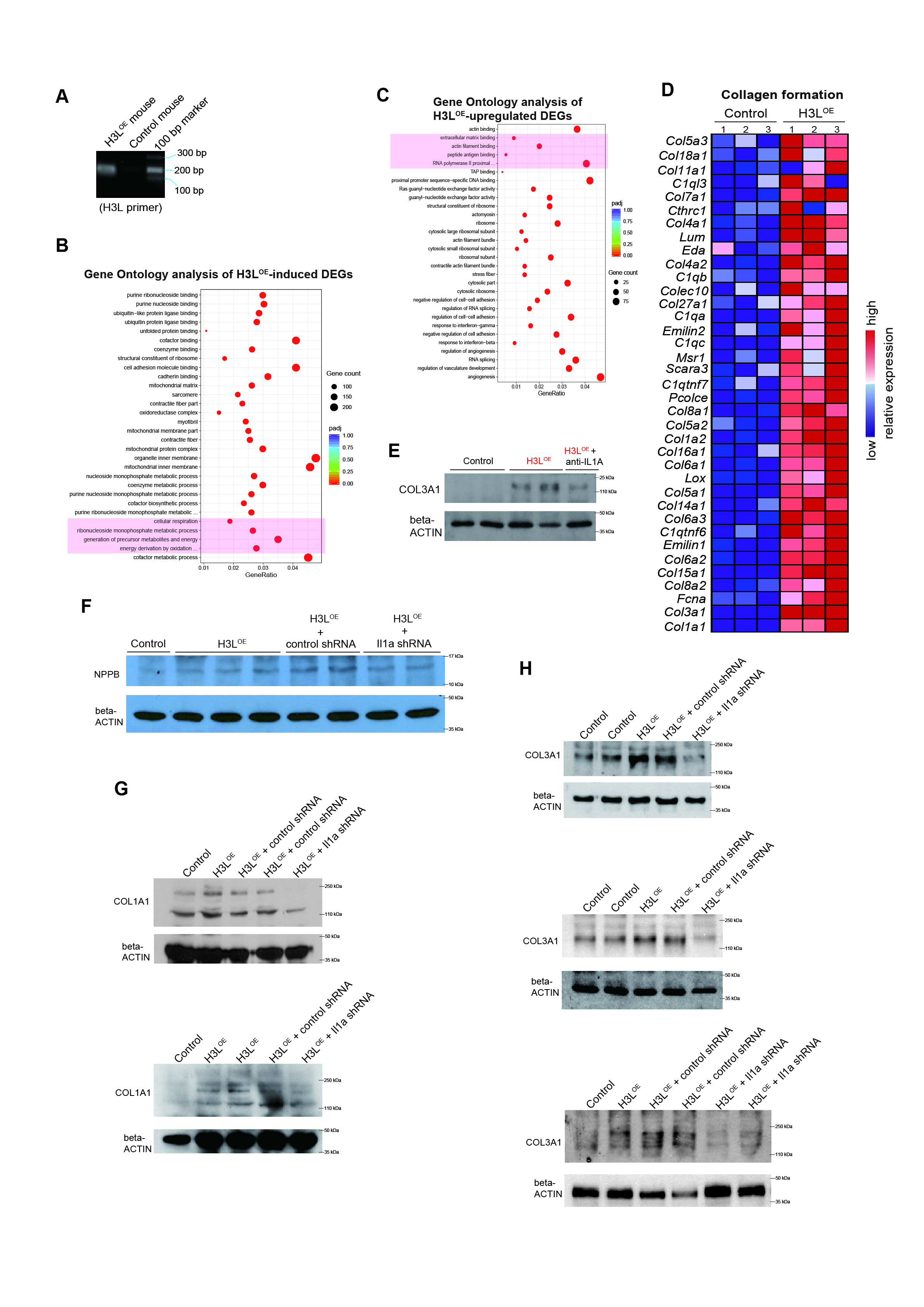

Supplement: Supplementary file 7 — supplemental figure 7 [file 41419_2024_6990_MOESM7_ESM.jpg]
