# Supplementary material for: Monkeypox virus protein H3L induces injuries in human and mouse
Source: Cell Death Dis. 2024 Aug 21;15(8):607. doi: 10.1038/s41419-024-06990-2 (PMC11339448; doi:10.1038/s41419-024-06990-2)
Supplement: Supplementary file 8 — supplemental figure legends [file 41419_2024_6990_MOESM8_ESM.docx]

**Supplemental Figure legends:**

**Fig. S1. RNA-seq analyses of Control, H3L^OE^, A29L^OE^, A35R^OE^ and I1L^OE^ hESCs.**

(A) Potential functions of four core proteins in Monkeypox virus (MPV).

(B) The scheme of establishing stable cell lines overexpressed with H3L, A29L, A35R, I1L and the control (blank virus control).

(C) Control, H3L^OE^, A29L^OE^, A35R^OE^ and I1L^OE^ hESCs cultured in mTesR1 medium. Scale bar, 100 µm.

(D) RT-qPCR showing the relative mRNA expression levels of H3L, A35R, I1L and A29L. *p<0.05.

(E) Western-blotting showing the protein expression levels of H3L, A29L, A35R and I1L. GAPDH was used as the internal control.

(F) Principal component analysis (PCA) of RNA-seq. Four biological replicates were applied.

(G-J) Kyoto Encyclopedia of Genes and Genomes (KEGG) analysis of DEGs induced by H3L (G), A29L (H), A35R (I) and I1L (J) in hESCs. DEGs, differentially expressed genes.

(K) DNase I treatment on genomic DNAs showing H3L overexpression-induced DNA instability.

(L) Western blots showing H3L overexpression increased the expression of cleaved caspase 3 and cleaved caspase 9.

(M) Flow cytometry quantified the percentage of cleaved caspase 3^+^ cells. *p<0.05.

(N) Flow cytometry quantified the percentage of Annexin V^+^ cells. *p<0.05.

**Fig. S2. H3L activates IL1A pathway**

(A) Signaling pathway analysis of differentially expressed genes induced by H3L in hESCs. Pathway analysis was run on Reactome.

(B) ChIP-seq revealed a potential binding of IRF4 on *IL1A* upstream distal enhancer. The black arrow showed the primer set of ChIP-qPCR by using specific IRF4 antibody.

**Fig. S3. RNA-seq analyses of Control and H3L^OE^ cardiac lineage cells derived from hESCs**

(A) Principal component analysis (PCA) analysis of RNA-seq. Two biological replicates for RNA-seq were applied on cardiac lineage cells derived from hESCs (on day 3 of cardiac differentiation).

(B) KEGG analysis of DEGs induced by H3L. DEGs, differentially expressed genes.

(C) GO analysis of differentially expressed genes induced by H3L.

(D) Heat map showing the H3L-downregulated genes, which control heart morphogenesis and cardiac development. FC, fold change.

(E) The *In vitro* 2D model of cardiac development. The cardiac lineage cells on day 3 (d3) were collected for RT-qPCR.

(F) Heat map showing the H3L-upregulated genes, which control DNA damage. FC, fold change.

(G) The read counts from RNA-seq showing *P53* expression change between Control and H3L^OE^ cells.

**Fig. S4. The expression and localization of H3L protein in hESCs**

(A) GESA analysis of differentially expressed genes induced by H3L. RNA-seq data was from Figure 3B.

(B) Heatmap showing differentially expressed genes involved in epigenetic regulation of gene expression. Data were from RNA-seq of Figure 3B.

(C) The FPKMs of RNA-seq in Figure 3B showing expression levels of epigenetic regulators. FPKM, fragments per kilobase of exon per million mapped fragments. Two biological replicates for RNA-seq.

(D-E) Immunostaining staining showing the expression and localization of H3L in hESCs (E). Green showed H3L protein expression (tag expression). Blue showed DAPI (nucleus). The fluorescence intensity (Mean) was calculated and quantified by Image J software (F). Scale bar, 20 µm. *p<0.05.

(F) Western-blot detecting protein expression level of Histone 3 in hESCs. ACTB, beta-actin.

**Fig. S5. ChIP-seq reveals H3L-bound genes**

(A-B) H3L was fused with myc-tag (A), followed with ChIP-seq analysis by using specific antibodies including anti-tag, anti-H3K4me3 and anti-H3K27me3 (B).

(C) The ChIP-seq reads across gene body. TSS, transcription start site. TES, transcription end site.

(D) The distributions of ChIP-seq binding reads.

(E) Gene Ontology (GO) analysis showing the top ranked GO terms of H3L-bound genes.

(F) Kyoto Encyclopedia of Genes and Genomes (KEGG) pathway analysis showing the top ranked KEGG pathways of H3L-bound genes.

**Fig. S6. H3L remodels chromatin and affects gene expression**

(A) Overlapping analysis of H3L ChIP-seq and RNA-seq. The overlapped genes bound by H3L and downregulated by H3L are associated with cardiac development cardiogenesis.

(B) Representative ChIP-seq peaks showing binding of H3L on mesodermal transcription factor (*MESP1*) in H3L^OE^ hESCs. Blue boxes highlighted binding regions of H3L or H3K27me3.

(C) ChIP-qPCR validation of H3L binding on promoters of *MESP1* in H3L^OE^ hESCs. *p<0.01 (vs. anti-IgG).

(D) ChIP-qPCR showing the binding changes of H3K4me3 and H3K27me3 on promoters of *MESP1* between Control and H3L^OE^ hESCs. *p<0.05 (H3L^OE^ cells vs. Control cells).

(E) Overlapping analysis of H3L ChIP-seq and RNA-seq. The overlapped genes bound by H3L and upregulated by H3L are involved in cell death.

(F) RNA-seq read counts of *IRF4* in hESCs (day 0). The RNA-seq data was from Figure 1G. *p<0.01.

(G) RT-qPCR showing relative expression level of *IRF4* in hESCs (day 0). *p<0.05.

(H) RNA-seq read counts and relative expression of *P53* in cardiac lineage cells (cardiac differentiation on day 3). The RNA-seq data was from Figure 1G.

(I) RT-qPCR showing relative expression level of *P53* in cardiac lineage cells (cardiac differentiation on day 3). *p<0.05.

**Fig. S7. H3L induces transcriptional perturbations in mouse heart tissues**

(A) RT-PCR followed with agarose gel running showing the expression of H3L in mouse heart tissues.

(B) Gene Ontology (GO) analysis of all differentially expressed genes induced by H3L.

(C) GO analysis of all upregulated genes induced by H3L.

(D) Heat map showing upregulated genes controlling collagen formation induced by H3L in mouse heart tissues.

(E) Western-blotting showing expression of COL3A1 in mouse heart tissues.

(F) Western-blotting showing expression of NPPB in mouse cardiomyocytes.

(G) Western-blotting showing expression of COL1A1 in mouse cardiomyocytes.

(H) Western-blotting showing expression of COL3A1 in mouse cardiomyocytes.
