# Supplementary material for: Monkeypox virus protein H3L induces injuries in human and mouse
Source: Cell Death Dis. 2024 Aug 21;15(8):607. doi: 10.1038/s41419-024-06990-2 (PMC11339448; doi:10.1038/s41419-024-06990-2)
Supplement: Supplementary file 9 — Supplementary Information [file 41419_2024_6990_MOESM9_ESM.docx]

**Supplementary Information**

**Monkeypox virus protein and DNA sequences：**

All protein sequences were from NCBI database: <https://www.ncbi.nlm.nih.gov/nuccore/ON563414>, Monkeypox virus isolate MPXV_USA_2022_MA001, complete genome (Gigante,C.M., et al.)

**I1L**, "DNA-binding core protein (Cop-I1L) I1L similar to Vaccinia virus strain Copenhagen I1L virosomal protein essential for virus multiplication":

**I1L** protein sequences: MAEFEDQLVFNSISARALKAYFTAKINEMVDELVTRKCPQKKKSQAKKPEVRIPVDLVKSSFVKKFGLCNYGGILISLINSLVENNFFTKNGKLDDTGKKELVLTDVEKRILNTIDKSSPLYIDISDVKVLAARLKRSATQFNFNGHTYHLENDKIEDLINQLVKDESIQLDEKSSIKDSMYVIPDELIDVLKTRLFRSPQVKDNIISRTRLYDYFTRVTKRDESSIYVILKDPRIASILSLETVKMGAFMYTKHSMLTNAISSRVDRYSKKFQESFYEDIAEFVKENERVNVSRVVECLTVPNITISSNTE

**A35R**, “EEV envelope glycoprotein, needed for formation of actin-containing microvilli and cell-to-cell spread of virion EEV membrane phosphoglycoprotein, C-type lectin-like domain (Cop-A33R) interacts with VAC A36R similar to Vaccinia virus strain Copenhagen A33R",

**A35R** protein sequences: MMTPENDEEQTSVFSATVYGDKIQGKNKRKRVIGLCIRISMVISLLSMITMSAFLIVRLNQCMSANKAAITDSAVAVAAASSTHRKVVSSTTQYDHKESCNGLYYQGSCYILHSDYKSFEDAKANCAAESSTLPNKSDVLTTWLIDYVEDTWGSDGNPITKTTSDYQDSDVSQEVRKYFCT

**A29L**, “IMV surface membrane 14 kDa fusion protein IMV surface protein, fusion protein (Cop-A27L) binding to cell surface heparan similar to Vaccinia virus strain Copenhagen A27L"

**A29L** protein sequences:

MDGTLFPGDDDLAIPATEFFSTKAAKNPETKREAIVKAYGDDNEETLKQRLTNLEKKITNITTKFEQIEKCCKRNDEVLFRLENHAETLRAAMISLAKKIDVQTGRHPYE"

**H3L**, "similar to Vaccinia virus strain Copenhagen H3L IMV heparin binding surface protein (Cop-H3L) IMVsurface membrane protein"

**H3L** protein sequences:

MAAVKTPVIVVPVIDRPPSETFPNVHEHINDQKFDDVKDNEVMQEKRDVVIVNDDPDHYKDYVFIQWTGGNIRDDDKYTHFFSGFCNTMCTEETKRNIARHLALWDSKFFTELENKNVEYVVIIENDNVIEDITFLRPVLKAIHDKKIDILQMREIITGNKVKTELVIDKDHAIFTYTGGYDVSLSAYIIRVTTALNIVDEIIKSGGLSSGFYFEIARIENEMKINRQIMDNSAKYVEHDPRLVAEHRFETMKPNFWSRIGTVAAKRYPGVMYTFTTPLISFFGLFDINVIGLIVILFIMFMLIFNVKSKLLWFLTGTFVTAFI

**I1L** coding DNA sequences (reverse translated from **I1L** protein sequences)：

ATGGCCGAGTTCGAGGACCAGCTGGTGTTCAACAGCATCAGCGCCAGGGCCCTGAAGGCCTACTTCACCGCCAAGATCAACGAGATGGTGGACGAGCTGGTGACCAGGAAGTGCCCCCAGAAGAAGAAGAGCCAGGCCAAGAAGCCCGAGGTGAGGATCCCCGTGGACCTGGTGAAGAGCAGCTTCGTGAAGAAGTTCGGCCTGTGCAACTACGGCGGCATCCTGATCAGCCTGATCAACAGCCTGGTGGAGAACAACTTCTTCACCAAGAACGGCAAGCTGGACGACACCGGCAAGAAGGAGCTGGTGCTGACCGACGTGGAGAAGAGGATCCTGAACACCATCGACAAGAGCAGCCCCCTGTACATCGACATCAGCGACGTGAAGGTGCTGGCCGCCAGGCTGAAGAGGAGCGCCACCCAGTTCAACTTCAACGGCCACACCTACCACCTGGAGAACGACAAGATCGAGGACCTGATCAACCAGCTGGTGAAGGACGAGAGCATCCAGCTGGACGAGAAGAGCAGCATCAAGGACAGCATGTACGTGATCCCCGACGAGCTGATCGACGTGCTGAAGACCAGGCTGTTCAGGAGCCCCCAGGTGAAGGACAACATCATCAGCAGGACCAGGCTGTACGACTACTTCACCAGGGTGACCAAGAGGGACGAGAGCAGCATCTACGTGATCCTGAAGGACCCCAGGATCGCCAGCATCCTGAGCCTGGAGACCGTGAAGATGGGCGCCTTCATGTACACCAAGCACAGCATGCTGACCAACGCCATCAGCAGCAGGGTGGACAGGTACAGCAAGAAGTTCCAGGAGAGCTTCTACGAGGACATCGCCGAGTTCGTGAAGGAGAACGAGAGGGTGAACGTGAGCAGGGTGGTGGAGTGCCTGACCGTGCCCAACATCACCATCAGCAGCAACACCGAGtaa

**A35R** coding DNA sequences (reverse translated from **A35R** protein sequences)：

ATGATGACCCCCGAGAACGACGAGGAGCAGACCAGCGTGTTCAGCGCCACCGTGTACGGCGACAAGATCCAGGGCAAGAACAAGAGGAAGAGGGTGATCGGCCTGTGCATCAGGATCAGCATGGTGATCAGCCTGCTGAGCATGATCACCATGAGCGCCTTCCTGATCGTGAGGCTGAACCAGTGCATGAGCGCCAACAAGGCCGCCATCACCGACAGCGCCGTGGCCGTGGCCGCCGCCAGCAGCACCCACAGGAAGGTGGTGAGCAGCACCACCCAGTACGACCACAAGGAGAGCTGCAACGGCCTGTACTACCAGGGCAGCTGCTACATCCTGCACAGCGACTACAAGAGCTTCGAGGACGCCAAGGCCAACTGCGCCGCCGAGAGCAGCACCCTGCCCAACAAGAGCGACGTGCTGACCACCTGGCTGATCGACTACGTGGAGGACACCTGGGGCAGCGACGGCAACCCCATCACCAAGACCACCAGCGACTACCAGGACAGCGACGTGAGCCAGGAGGTGAGGAAGTACTTCTGCACCtaa

**A29L** coding DNA sequences (reverse translated from **A29L** protein sequences)：

ATGGACGGCACCCTGTTCCCCGGCGACGACGACCTGGCCATCCCCGCCACCGAGTTCTTCAGCACCAAGGCCGCCAAGAACCCCGAGACCAAGAGGGAGGCCATCGTGAAGGCCTACGGCGACGACAACGAGGAGACCCTGAAGCAGAGGCTGACCAACCTGGAGAAGAAGATCACCAACATCACCACCAAGTTCGAGCAGATCGAGAAGTGCTGCAAGAGGAACGACGAGGTGCTGTTCAGGCTGGAGAACCACGCCGAGACCCTGAGGGCCGCCATGATCAGCCTGGCCAAGAAGATCGACGTGCAGACCGGCAGGCACCCCTACGAGtaa

**H3L** coding DNA sequences (reverse translated from **H3L** protein sequences)：

ATGGCCGCCGTGAAGACCCCCGTGATCGTGGTGCCCGTGATCGACAGGCCCCCCAGCGAGACCTTCCCCAACGTGCACGAGCACATCAACGACCAGAAGTTCGACGACGTGAAGGACAACGAGGTGATGCAGGAGAAGAGGGACGTGGTGATCGTGAACGACGACCCCGACCACTACAAGGACTACGTGTTCATCCAGTGGACCGGCGGCAACATCAGGGACGACGACAAGTACACCCACTTCTTCAGCGGCTTCTGCAACACCATGTGCACCGAGGAGACCAAGAGGAACATCGCCAGGCACCTGGCCCTGTGGGACAGCAAGTTCTTCACCGAGCTGGAGAACAAGAACGTGGAGTACGTGGTGATCATCGAGAACGACAACGTGATCGAGGACATCACCTTCCTGAGGCCCGTGCTGAAGGCCATCCACGACAAGAAGATCGACATCCTGCAGATGAGGGAGATCATCACCGGCAACAAGGTGAAGACCGAGCTGGTGATCGACAAGGACCACGCCATCTTCACCTACACCGGCGGCTACGACGTGAGCCTGAGCGCCTACATCATCAGGGTGACCACCGCCCTGAACATCGTGGACGAGATCATCAAGAGCGGCGGCCTGAGCAGCGGCTTCTACTTCGAGATCGCCAGGATCGAGAACGAGATGAAGATCAACAGGCAGATCATGGACAACAGCGCCAAGTACGTGGAGCACGACCCCAGGCTGGTGGCCGAGCACAGGTTCGAGACCATGAAGCCCAACTTCTGGAGCAGGATCGGCACCGTGGCCGCCAAGAGGTACCCCGGCGTGATGTACACCTTCACCACCCCCCTGATCAGCTTCTTCGGCCTGTTCGACATCAACGTGATCGGCCTGATCGTGATCCTGTTCATCATGTTCATGCTGATCTTCAACGTGAAGAGCAAGCTGCTGTGGTTCCTGACCGGCACCTTCGTGACCGCCTTCATCtaa

**Key resource table**

| **Reagent or Resource** | **Source** | **Identifier** |
| --- | --- | --- |
| **Antibodies** | | |
| myc-Tag (9B11) Mouse mAb | Cell Signaling Technology | Cat: 2276S,  RRID: AB_331783 |
| Phospho-Histone H2A.X (Ser139) (20E3) Rabbit mAb | Cell Signaling Technology | Cat: 9718,  RRID: AB_2118009 |
| IgG Isotype Control | R&D system | MAB002 |
| IgG Isotype Control | Millipore | MAGNARIP01 |
| Cardiac Troponin T | Thermo Fisher | Cat: MS-295-P,  RRID: AB_61806 |
| APC goat anti-mouse IgG | BD Biosciences | Cat: 550826,  RRID: AB_398465 |
| Oct-4A (C30A3) Rabbit mAb | Cell Signaling Technology | Cat: 2840,  RRID: AB_2167691 |
| Goat anti-mouse Alexa Fluor 488 | Invitrogen | Cat: A28175  RRID:AB_2534069 |
| Goat anti-rabbit Alexa Fluor 555 | Invitrogen | Cat: A27039  RRID: AB_2536100 |
| Brachyury (D2Z3J) Rabbit mAb | Cell Signaling Technology | Cat: 81694,  RRID: AB_2799983 |
| Histone H3 (1B1B2) Mouse mAb | Cell Signaling Technology | Cat: 14269,  RRID: AB_2756816 |
| Tri-Methyl-Histone H3 (Lys4) (C42D8) Rabbit mAb | Cell Signaling Technology | Cat: 9751,  RRID:  AB_2616028 |
| Tri-Methyl-Histone H3 (Lys27) (C36B11) Rabbit mAb | Cell Signaling Technology | Cat: 9733 |
| ANP Polyclonal Antibody | Thermos Fisher | Cat: PA5-29559,  RRID: AB_2547035 |
| BNP Polyclonal Antibody | Thermos Fisher | Cat: PA5-96084,  RRID: AB_2807886 |
| p53 Monoclonal Antibody (BP53-12) | Invitrogen | Cat: MA1-19055,  RRID: AB_1077416 |
| IL-1 alpha Polyclonal Antibody | Thermos Fisher | Cat: PA5-96081,  RRID: AB_2807883 |
| GAPDH (14C10) Rabbit mAb | Cell Signaling Technology | Cat: 2118,  RRID: AB_561053 |
| IRF4 Monoclonal Antibody (3E4) | eBioscience | Cat: 14-9858-82,  RRID: AB_10804654 |
| Wheat Germ Agglutinin (WGA) | Thermos Fisher | Cat: W11261, RRID: AB_2334867 |
| COL1A1 Polyclonal Antibody | Thermos Fisher | Cat: PA5-29569,  RRID: AB_2547045 |
| COL3A1 Polyclonal Antibody | Thermos Fisher | Cat: PA5-27828,  RRID: AB_2545304 |
| Anti-beta Actin antibody | Abcam | Cat: ab8227, RRID: AB_2305186 |
|  |  |  |
| **Critical commercial reagents** |  | |
| Magna ChIP™ A/G Chromatin Immunoprecipitation kit | Millipore | 17-10085 |
| In Situ Cell Death Detection Kit, Fluorescein | Roche | 11684795910 |
| Rock inhibitor Y27632 | STEMCELL Technologies | 72304 |
| SYBR Premix Ex Taq | Takara Bio | RR420A |
| 1st strand cDNA Synthesis Kit | Takara Bio | 6110A |
| mTesR1 medium | STEMCELL Technologies | 85850 |
| ReLeSR™ Passaging Reagent | STEMCELL Technologies | 100-0484 |
| truChIP® Chromatin Shearing Kit | Covaris | 520154 |
| STEMdiff™ Cardiomyocyte Differentiation Kit | STEMCELL Technologies | 05010 |
| STEMdiff™ Cardiomyocyte Maintenance Kit | STEMCELL Technologies | 05020 |
| STEMdiff™ Cardiomyocyte Dissociation Kit | STEMCELL Technologies | 05025 |
| CryoStor® CS10 | STEMCELL Technologies | 07930 |
| Matrigel® hESC-Qualified Matrix, LDEV-free | Corning | 354277 |
| miRNeasy mini kit | Qiagen | 217004 |
| Human IL-1A ELISA Kit | Invitrogen | BMS243-2 |
| Mouse IL-1 alpha ELISA | RayBiotech | ELM-IL1a |
| IFN beta Human ELISA Kit | Invitrogen | 414101 |
| IFN alpha Human ELISA Kit | Invitrogen | BMS216 |
| IFN gamma Human ELISA Kit | Invitrogen | EHIFNG |
| RNeasy Micro Kit | Qiagen | 74004 |
| **Experimental Models: Cell Lines** | | |
| Human ESC H9 cell line | Donated by Dr. Xiaohong Li in Guangdong Provincial People’s Hospital | N/A |
|  |  |  |
| **Recombinant DNA** | | |
| pLVX-puro-EF1a-3X Myc (modified in lab) | Addgene | #51394 |
| psPAX2 | Addgene | #12260 |
| pMD2.G | Addgene | #12259 |
| pLKO.1-TRC-puro vector | Addgene | #10878 |
| lentiCRISPRv2-puro vector | Addgene | #52961 |
| **Software and Algorithms** | | |
| Image J | National Institutes of Health | https://imagej.nih.gov/ij/ |
| Flow Jo | Becton, Dickinson and Company | https://www.flowjo.com/ |
| ZEISS ZEN microscope software for light microscopy systems | ZEISS | https://www.zeiss.com/microscopy/us/products/microscope-software/zen-lite.html |
| THE GENE ONTOLOGY RESOURCE | N/A | http://geneontology.org/ |
| Reactome Pathway Database | N/A | https://reactome.org/ |
| GraphPad Prism | GraphPad | https://www.graphpad.com/ |
|  |  |  |
