# Supplementary material for: Monkeypox virus protein H3L induces injuries in human and mouse
Source: Cell Death Dis. 2024 Aug 21;15(8):607. doi: 10.1038/s41419-024-06990-2 (PMC11339448; doi:10.1038/s41419-024-06990-2)

Figure 1F Figure S1E

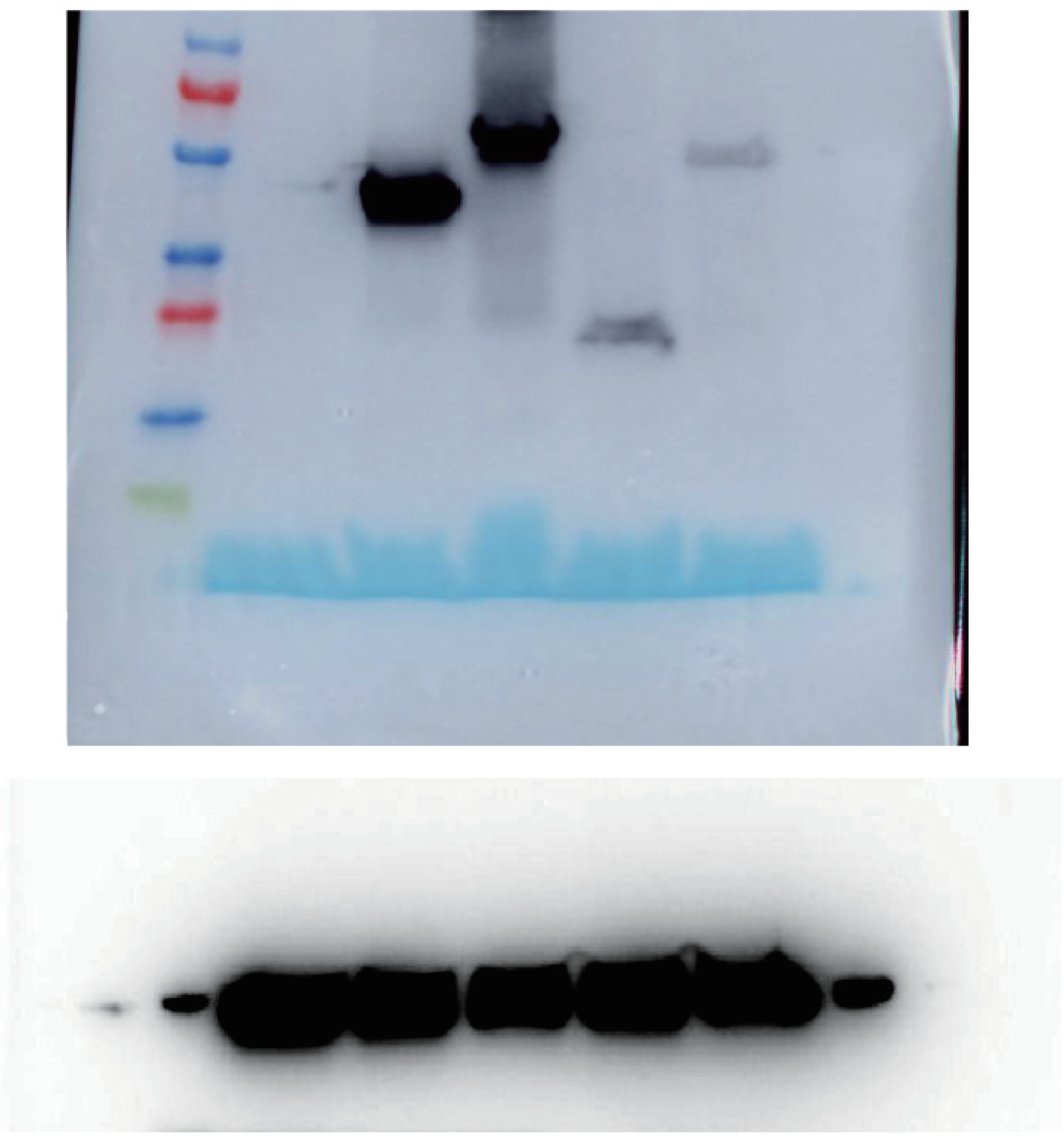

Figure 4P

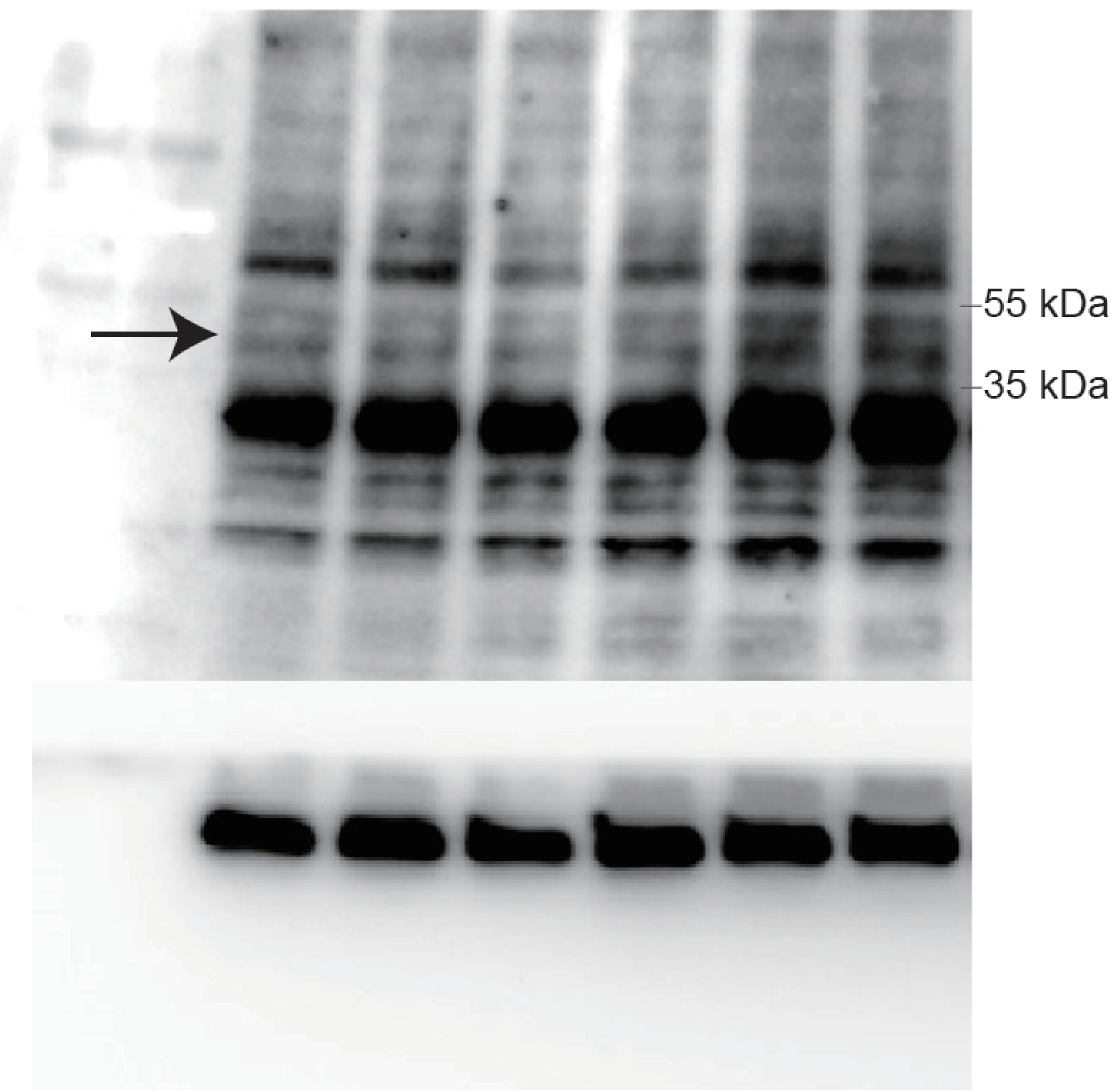

Figure 4R

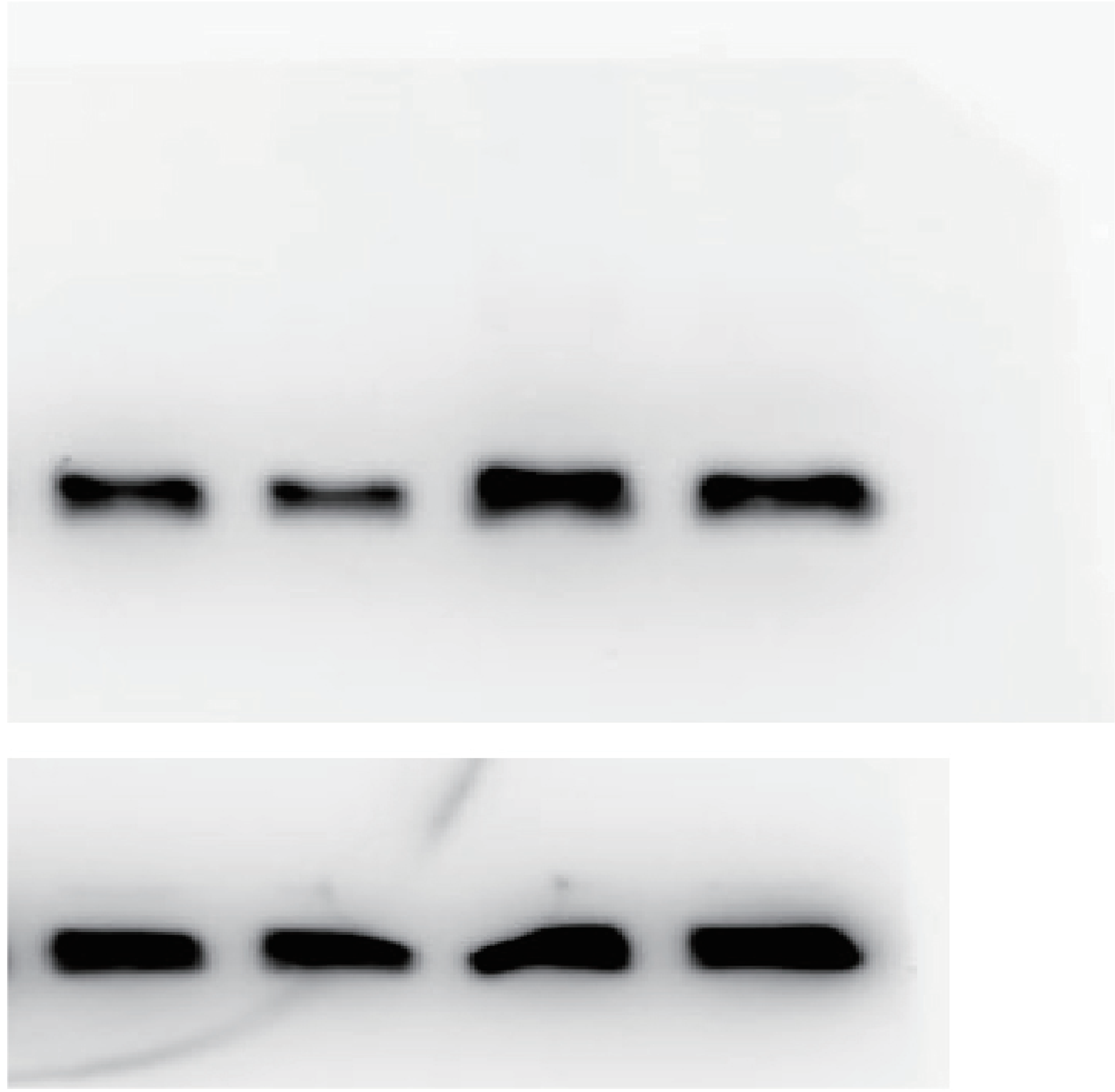

Figure 4T

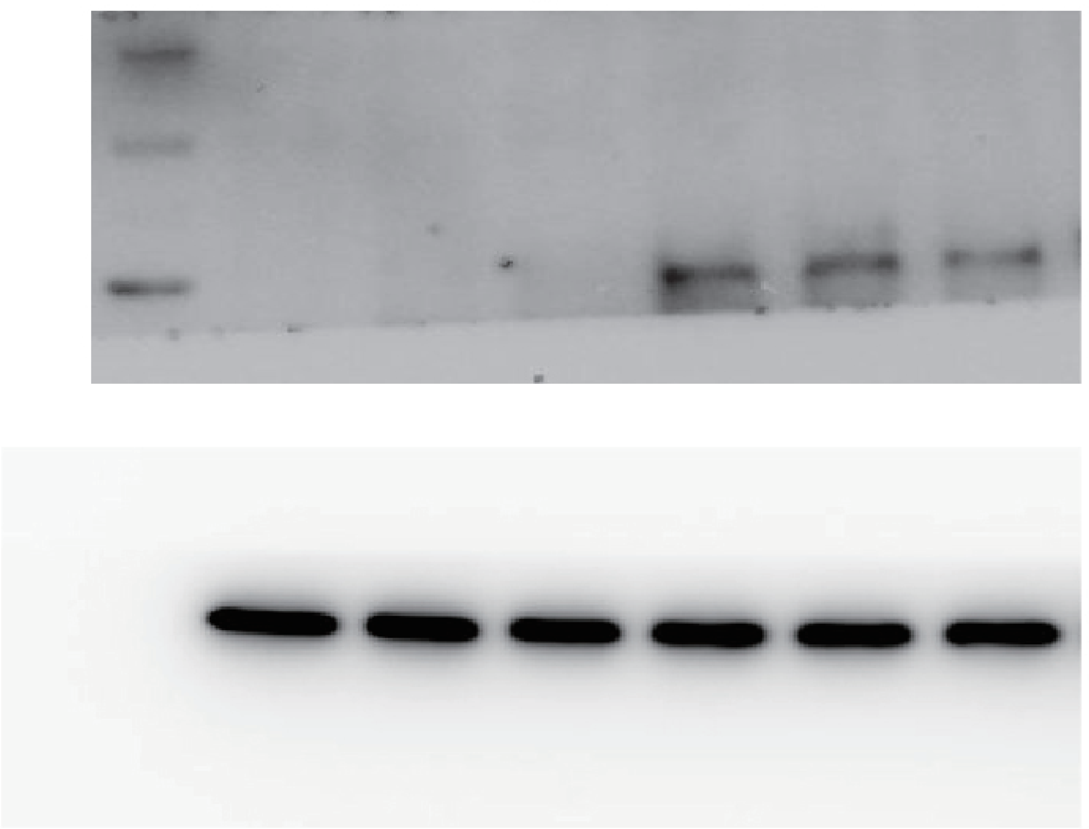

Figure S4F

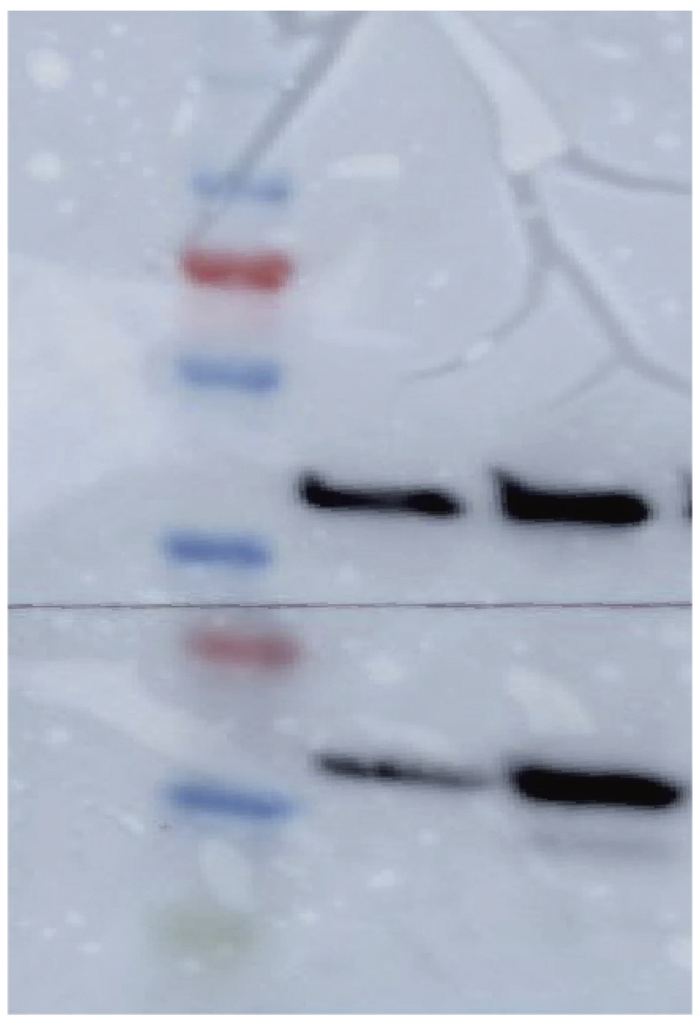

Figure S1L

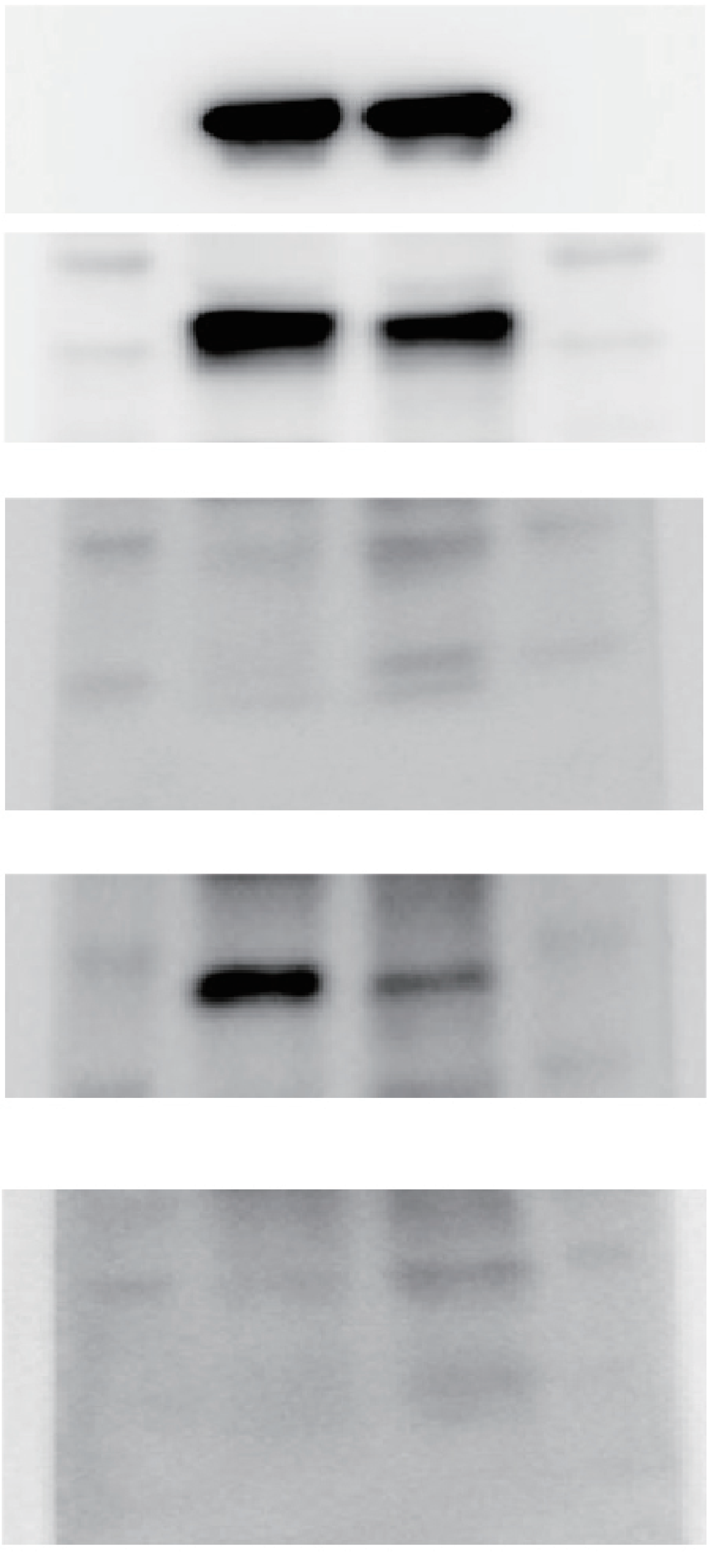

Figure 7C

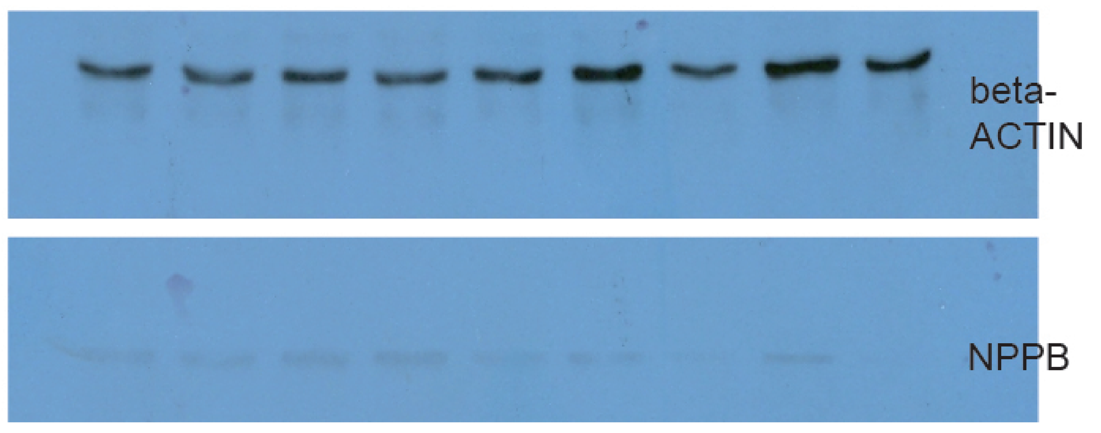

Figure S7F

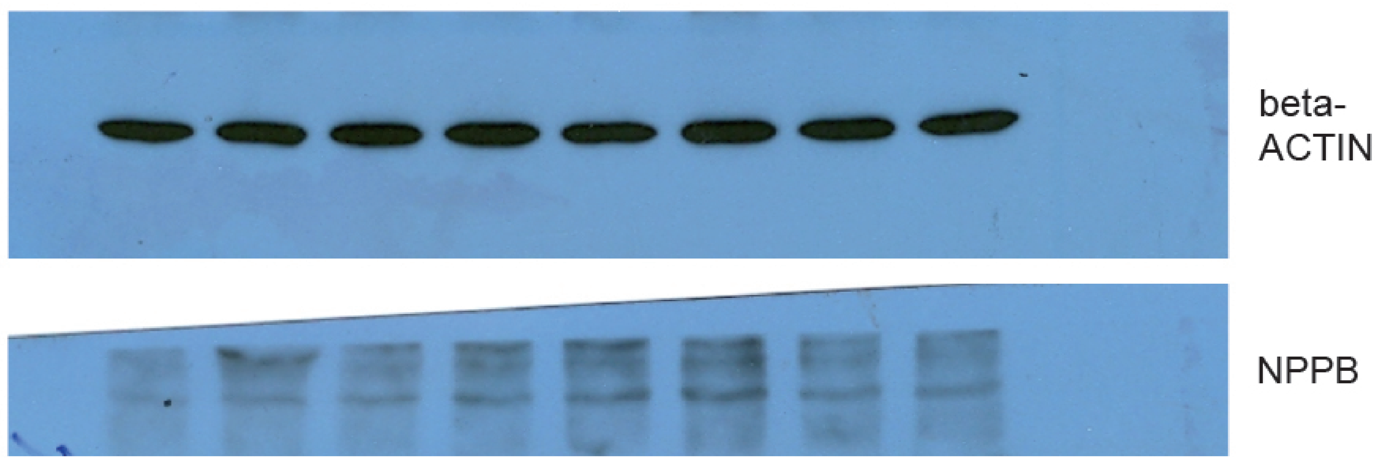

Figure 7D

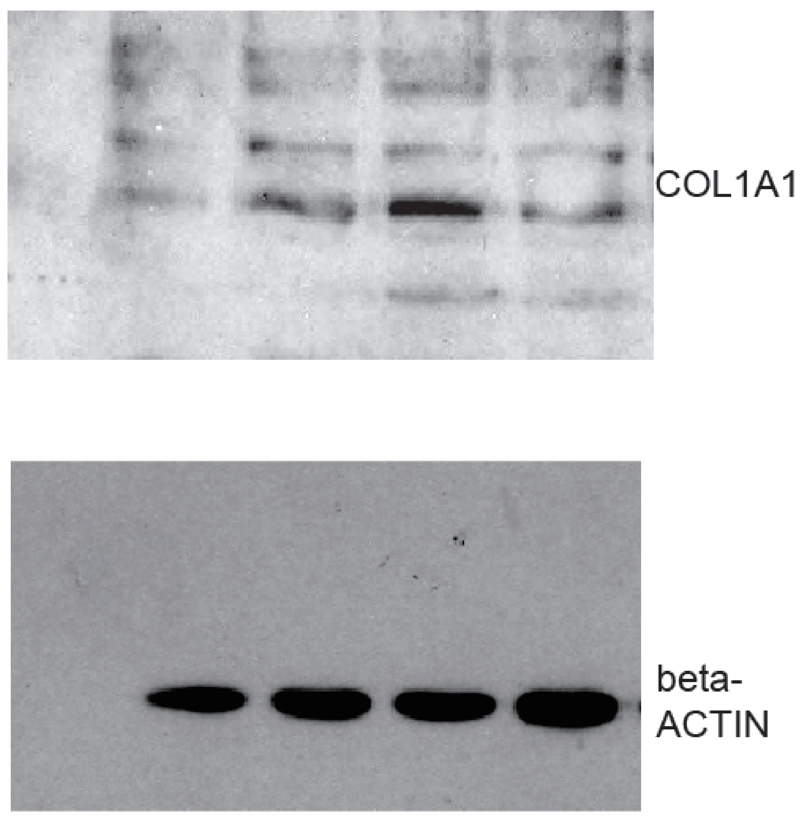

Figure 7E (Figure S7H)

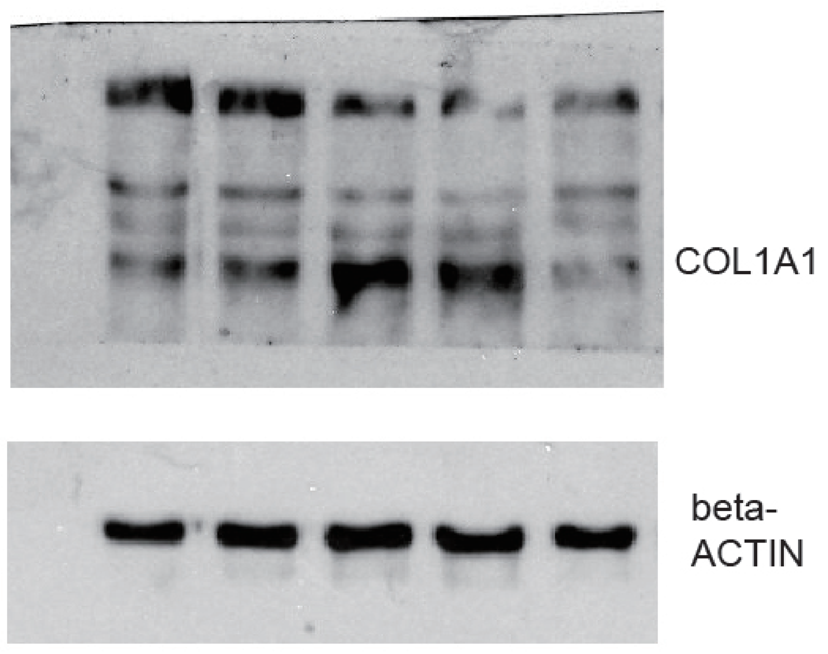

Figure S7G

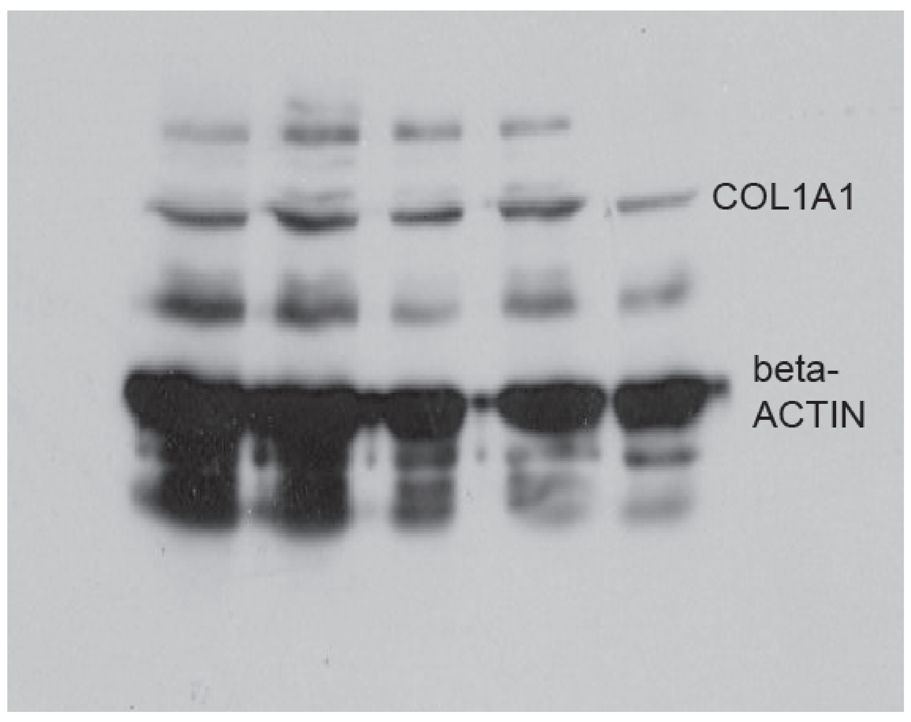

Figure S7H

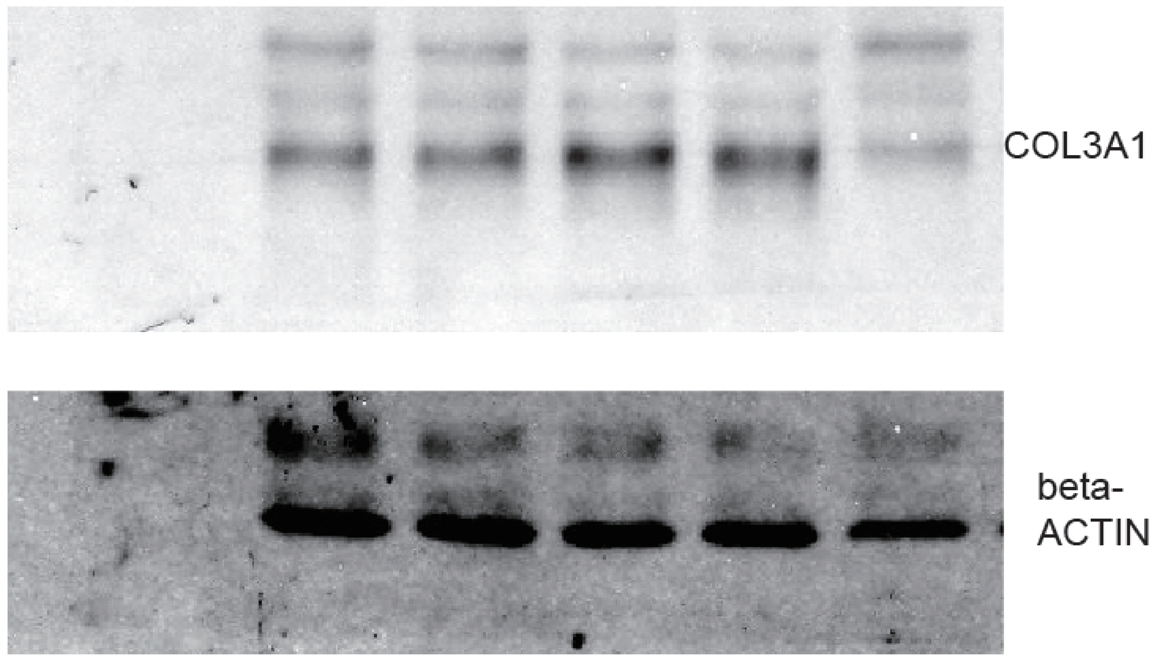

Figure S7G

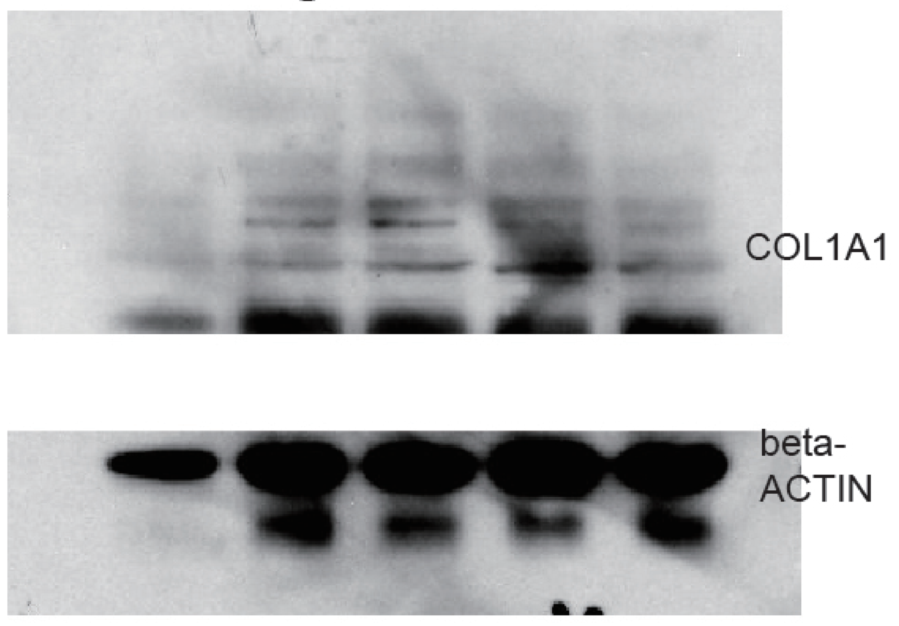

Figure S7H

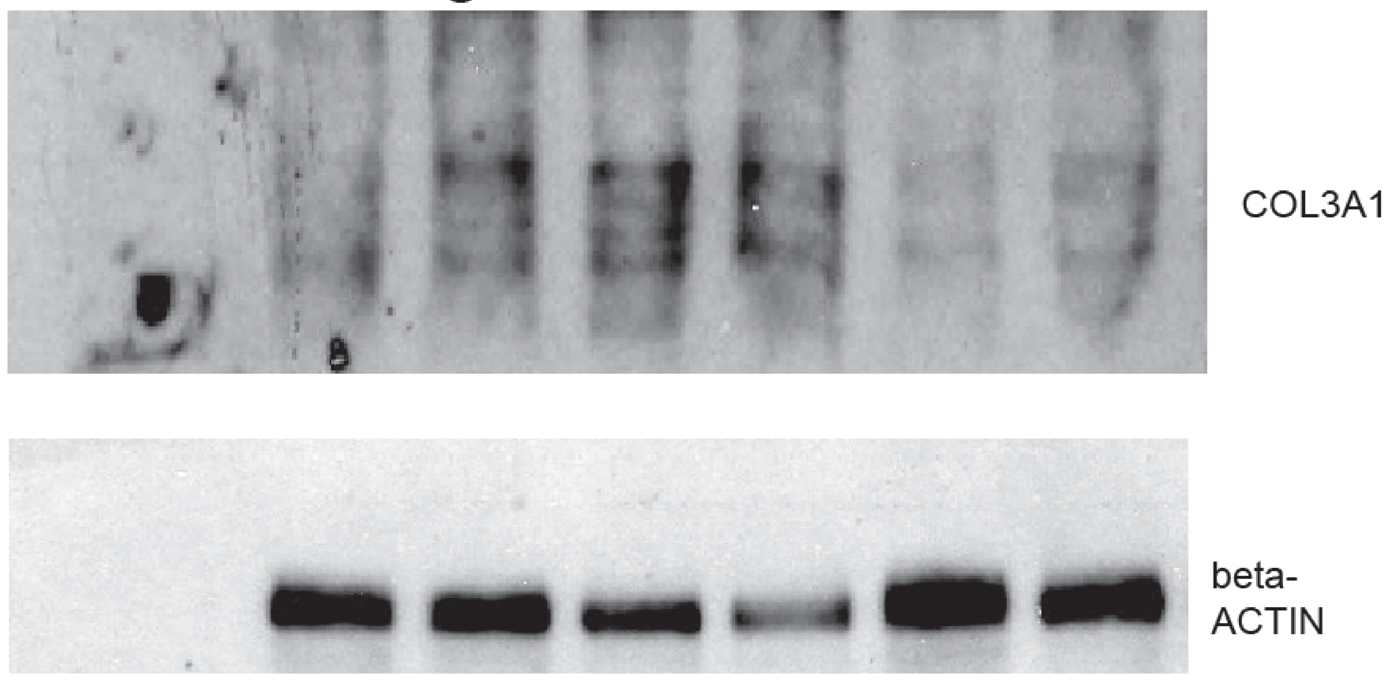

Supplement: Supplementary file 15 — Supplemental material-Western blots [file 41419_2024_6990_MOESM15_ESM.pdf]
